# CBP/p300 Bromodomain Inhibitor–I–CBP112 Declines Transcription of the Key ABC Transporters and Sensitizes Cancer Cells to Chemotherapy Drugs

**DOI:** 10.3390/cancers13184614

**Published:** 2021-09-14

**Authors:** Magdalena Strachowska, Karolina Gronkowska, Sylwia Michlewska, Agnieszka Robaszkiewicz

**Affiliations:** 1 Department of General Biophysics, Faculty of Biology and Environmental Protection, University of Lodz, Pomorska 141/143, 90-236 Lodz, Poland; magdalena.strachowska@edu.uni.lodz.pl (M.S.); karolina.gronkowska@edu.uni.lodz.pl (K.G.); 2 Laboratory of Microscopic Imaging and Specialized Biological Techniques, Faculty of Biology and Environmental Protection, University of Lodz, Banacha 12/16, 90-237 Lodz, Poland; sylwia.michlewska@biol.uni.lodz.pl

**Keywords:** I-CBP112, CBP/p300 bromodomain inhibitor, ATP-binding cassette transporters (ABC), lysine-specific demethylase 1A (LSD1), histone modifications, anticancer drugs

## Abstract

**Simple Summary:**

Despite tremendous advances in cancer treatment, chemotherapy remains the first-line choice in many tumor types. The action of numerous chemotherapy drugs is limited by the occurrence of ABC proteins in cancer cell membranes, which remove medicines from cell compartments. In this paper, we show that one of bromodomain inhibitors, namely I-CBP112, was capable of repressing genes that are responsible for multidrug resistance in all three studied cancer cell lines. CBP/p300 bromodomain inhibitor allows for the higher drug accumulation inside cells and considerably potentiated drug effects. At the molecular level, I-CBP112 caused rearrangement of chromatin at the ABC gene promoters by inducing recruitment of LSD1, which removes transcription-promoting histone marks. I-CBP112 emerges as a promising compound to overcome ABC-dependent cancer drug resistance.

**Abstract:**

The high expression of some ATP-binding cassette (ABC) transporters is linked to multidrug resistance in cancer cells. We aimed to determine if I-CBP112, which is a CBP/p300 bromodomain inhibitor, altered the vulnerability of the MDA-MB-231 cell line to chemotherapy drugs, which are used in neoadjuvant therapy in patients with triple negative breast cancer (TNBC). MDA-MB-231 cells represent TNBC, which is negative for the expression of estrogen and progesterone receptors and HER2 protein. An I-CBP112-induced decrease in the expression of all the studied ABCs in the breast, but also in the lung (A549), and hepatic (HepG2) cancer cell lines was associated with increased accumulation of doxorubicin, daunorubicin, and methotrexate inside the cells as well as with considerable cell sensitization to a wide range of chemotherapeutics. Gene promoters repressed by I-CBP112 in MDA-MB-231 cells, such as ABCC1 and ABCC10, were characterized by enhanced nucleosome acetylation and, simultaneously, by considerably lower trimethylation in the transcription-promoting form of H3K4me3. The CBP/p300 bromodomain inhibitor induced the recruitment of LSD1 to the gene promoters. The inhibition of this demethylase in the presence of I-CBP112 prevented the repression of ABCC1 and ABCC10 and, to a considerable extent, cancer cells’ sensitization to drugs. In conclusion, the CBP/p300 bromodomain inhibitor I-CBP112 can be considered as a potent anti-multidrug-resistance agent, capable of repressing key ABC transporters responsible for drug efflux in various cancer types.

## 1. Introduction

Bromodomain inhibitors, having emerged as a promising class of anticancer drugs over the last decade, are now being tested in clinical trials for the treatment of various types of cancers. These small-molecule inhibitors target bromodomains (BRDs), which are evolutionarily conserved protein–protein interaction modules characterized by a bundle of four α-helices linked to each other by loop segments of variable length [1]. This deep, largely hydrophobic pocket recognizes acetylated lysine residues on histone and, to a lesser extent, non-histone proteins. BRD-containing proteins represent a group of chromatin readers that are capable of histone recognition, further modifications, and the regulation of transcriptional machinery by the recruitment of molecular partners such as components of the transcriptional complex and positive elongation factor (P-TEFb) [2,3]. Some of these proteins act as transcription factors, such as the bromodomain and extraterminal domain (BET) family members BRD2-4 and BRDT, whereas many others serve as transcription activators, including histone acetyltransferases (EP300, GCN5, and CREBBP), methyltransferases (MLL and ASH1L), and SWI/SNF components (BRG1/SMARCA4).

In our previous reports, we showed that BRG1 and nucleosome acetylation were enriched at the promoters of some ATP-binding cassette transporters (ABC transporters), such as ABCC4, ABCC5, ABCC10, and ABCG2, in the triple-negative breast cancer cell line MDA-MB-231. Membrane-bound ABC proteins are involved in ATP-dependent cellular transport across biological barriers. In addition to protecting cells from drugs and environmental toxins, these proteins play a role in the efflux of cholesterol and steroid hormones, vitamins, cytokines, and chemokines, as well as prostaglandins, thereby regulating intracellular processes [4]. The ABCC subgroup of this family, called multidrug-resistance proteins (MDRs), is particularly known for its involvement in cancer cell resistance to a wide range of anticancer drugs due to its low substrate specificity. However, proteins from other subfamilies, such as ABCB (ABCB1, glycoprotein-P or multidrug protein (MRP)) and ABCG (ABCG2, breast cancer resistance protein (BRCP)), also contribute to cancer irresponsiveness to chemotherapy. According to Chelamalla, 45% of cancers are resistant to standard anticancer drugs, and even initially responsive tumors develop resistance during the following cycles of chemotherapy [5], which is still leading among other anticancer approaches. For example, chemotherapy before surgery, known as neoadjuvant chemotherapy, is a frequent choice for treatment in women diagnosed with triple-negative breast cancer, since it leads to a pathologic complete response and improves disease-free survival and overall survival [6,7]. This tumor type accounts for about 10–15% of all breast tumors and does not respond to hormonal or HER2-targeted therapy, and the lack of estrogen, progesterone, and HER2 receptors limits the treatment options to a combination of surgery, radiation therapy, and chemotherapy. This has triggered intense interest in finding new medications that can treat this kind of breast cancer or improve current approaches.

Knowing that BRG1 forms a functional regulatory unit with EP300 and that acetyltransferase is responsible for the BRG1-driven transcriptional activity of some cell-cycle-dependent genes, we aimed to determine whether the specific acetyl-lysine competitive protein–protein interaction inhibitor I-CBP112, which targets the bromodomain of the two closely related and highly homologous acetyltransferases CBP/EP300, could decrease the expression of multidrug-resistance proteins in the triple-negative MDA-MD-231 breast cancer cell line and thereby enhance the toxicity of anticancer drugs. I-CBP112 was also shown to enhance nucleosome acetylation, probably by allosterically activating CBP/EP300, through bromodomain interactions [8]. The combination of I-CBP112 and A-485, targeting distinct parts of CBP/EP300—the bromodomain and histone acetyltransferase domain, respectively—arrested the proliferation of the prostate cancer cells as well as suppressed androgen-dependent and pro-oncogenic prostate genes, such as KLK3 (PSA) and c-Myc, which was followed by a strong reduction in p300 chromatin occupancy at their gene promoters [9]. In cultures of human and mouse leukemic cell lines, I-CBP112 impaired aberrant self-renewal and, interestingly, increased the sensitization of cells to the activity of the BET bromodomain inhibitor JQ1 and doxorubicin [10].

These examples indicate that I-CBP112 is capable of regulating the transcription of cancer genes and increasing cancer cells’ vulnerability to, at least some, anticancer drugs. Given the above-mentioned findings, we studied the impact of the CBP/EP300 bromodomain inhibitor on the expression of ABC transporters, which are highly transcribed in the breast cancer cell line MDA-MB-231 and functionally linked to cancer multidrug resistance. We also assayed the possible impact of ABC-transporter modulation on the accumulation and toxicity of anticancer drugs. We tested a relatively large panel of therapeutics, which represents various mechanisms of anticancer activity, to find the most efficient combinations with I-CBP112.

## 2. Materials and Methods

### 2.1. Materials

A549 and HepG2 cell lines were purchased from ATCC, whereas MDA-MB-231 from Sigma Aldrich. DMEM high glucose w/L-glutamine w/sodium pyruvate, fetal bovine serum and antibiotics (penicillin and streptomycin) were from Biowest (CytoGen, Zgierz, Poland). L15 Medium, oligonucleotides for ChIP-real-time PCR, KAPA SYBR^®^ FAST Universal 2x, resazurin sodium salt, probenecid, daunorubucun hydrochloride, methotrexate, bleomycin sulfate (*Streptomyces verticillus*), Nunc^®^ MicroWell™ 384 well optical bottom plates were from Sigma Aldrich (Poznan, Poland). Etoposide, doxorubicin hydrochloride, cisplatin, paclitaxel, SP2509 (iLSD1), and PBIT (iKDM5B) were from Cayman Chemical (Biokom, Janki/Warsaw, Poland), Nunc™ Lab-Tek™ chamber slides were ordered also in Biokom, Janki/Warsaw, Poland. Anti-CDK4 (sc-23896), anti-PCNA (sc-56) and anti-CCNE (sc-247) antibodies were purchased from Santa Cruz Biotechnology (AMX, Lodz, Poland). The high-capacity cDNA reverse transcription kit, SuperSignal™ West Pico chemiluminescent substrate, TRI Reagent™, PageRuler™ pre-stained protein ladder (10 to 180 kDa), Pierce™ protease inhibitor tablets (EDTA-free; PIC), goat anti-mouse IgG (H + L) secondary antibody, HRP (32430), Texas red-X phalloidin, ProLong™ diamond antifade mountant, SlowFade™ glass soft-set antifade mountant (with DAPI), anti-MRP5 (ABCC5) polyclonal antibody (PA5102678), anti-MRP10 (ABCC10) polyclonal antibody (PA5101678), TaqMan™ Universal Master Mix II, TaqMan™ gene expression assays (FAM-MGB/20X) for ABCG2 (Hs01053790_m1), ABCC10 (Hs01056200_m1), ABCC5 (Hs00981089_m1), ABCB1 (Hs00184500_m1), ABCC1 (Hs01561483_m1), ABCC2 (Hs00960489_m1), ABCC3 (Hs00978452_m1), ABCC4 (Hs00988721_m1), GAPDH (Hs02786624_g1), ACTB (Hs01064292_g1) were from Thermofisher Scientific (Thermofisher Scientific, Warsaw, Poland). Anti-ABCB1 (E1Y7B) rabbit mAb (#13342), anti-ABCC1 (D7O8N) rabbit mAb (#14685), ABCG2 (D5V2K) XP^®^ rabbit mAb (#42078), anti-p300 (D2 × 6N) rabbit mAb (#54062), anti-ABCC4 (D2Q2O) rabbit mAb (#12705), anti-ABCC3 (D8V8J) rabbit mAb (#39909), anti-LSD1 (#2139), anti-histone H3 (#4620; ChIP grade), anti-H3K27ac (#4353), anti-H3K4me3 (#9751), normal rabbit IgG (#2729), anti-histone H3 (1B1B2; for Western blot) mouse mAb (#14269), anti-mouse IgG (H + L), F (ab′)2 fragment (PE conjugate) (#59997), anti-rabbit IgG (H + L), F (ab′)2 fragment (Alexa Fluor^®^ 488 Conjugate) (#4412), anti-rabbit IgG, HRP-linked antibody (#7074) were from Cell Signaling Technologies (LabJOT, Warsaw, Poland). The FITC annexin V apoptosis detection kit with propidium iodide was purchased from BioLegend (BioCourse.pl, Katowice, Poland), ApoTox-Glo™ triplex assay was from Promega (Promega, Warsaw, Poland).

### 2.2. Cell Culture and Treatment with Inhibitors

A549 and HepG2 were cultured in DMEM supplemented with 10% FBS and penicillin/streptomycin (50 U/mL and 50 µg/mL, respectively) in 5% CO_2_. Initially, MDA-MB-231 cells were cultured in F15 medium supplemented with 15% FBS and penicillin/streptomycin (50 U/mK and 50 µg/mL, respectively) without CO_2_ equilibration. After 5 passages, the cells were adapted to grow in DMEM supplemented with 10% FBS and penicillin/streptomycin (50 U/mL and 50 µg/mL, respectively) in 5% CO_2_.

I-CBP112 was added to cells 72 h prior to analysis or treatment with anticancer drugs, which were administrated to cells for another 4–48 h (depending on the tested parameters).

### 2.3. Real-Time PCR

For mRNA quantification, the total RNA was extracted with TRI Reagent™ and reverse-transcribed with a high-capacity cDNA reverse transcription kit (Thermofisher Scientific), and the expression of selected genes was measured with TaqMan™ gene expression sssays and the TaqMan™ universal master mix II (Thermofisher Scientific), according to the protocol provided by the manufacturer (polymerase activation: 95 °C, 10 min; PCR cycles: denaturation at 95 °C, 15 s; annealing and extension at 60 °C, 1 min). ACTB and GAPDH (HSKG) were used for normalization, and the ratio between the studied gene and HSKG was assumed to be 1 for control (untreated) cells.

### 2.4. Western Blot

For protein visualization, cell lysates collected in RIPA buffer (supplemented with 1 mM PMSF, 1× protease inhibitor) were separated by SDS–PAGE, transferred to a nitrocellulose membrane, and stained with primary antibodies (1:5000) at 4 °C overnight. After subsequent staining with HRP-conjugated secondary antibodies (1:10,000 for anti-rabbit and 1:500 for anti-mouse antibodies; room temperature; 2 h), the signal was developed using the SuperSignal™ west pico chemiluminescent substrate and acquired with a ChemiDoc-IT2 (UVP, Meranco, Poznan, Poland). Histone H3 was used as the loading control. All the whole western blot figures can be found in the supplementary materials. 

### 2.5. Confocal Microscopy

For the confocal imaging of ABC proteins, cells were seeded and treated with I-CBP112 on a Nunc™ Lab-Tek™ chamber slide, fixed with a 1% formaldehyde solution in PBS at room temperature for 15 min, washed 3× with PBS, and permeabilized and blocked with 1% FBS solution in PBS with 0.1% TritonX-100 at room temperature for at least 1 h. Primary antibodies (1:400) were added in 1% BSA solution in PBS with 0.1% TritonX-100 and incubated at 4 °C overnight. Secondary antibody (1:400) was added in 1% BSA solution in PBS with 0.1% TritonX-100 at room temperature for 2 h. After washing, the slides were mounted with SlowFade™ glass soft-set antifade mountant (with DAPI). TCS SP8 (Leica Microsystems, Germany) with a 63×/1.40 objective (HC PL APO CS2, Leica Microsystems, Germany) was used for sample visualization. The samples were imaged with the following wavelength values for excitation and emission: 485 and 500–550 nm for Alexa Fluor^®^ 488 and 405 and 430–480 nm for DAPI. The average fluorescence was calculated using at least 100 single cells for each sample. The fluorescence intensity was determined in arbitrary units (a.u.) with Leica Application Suite X (LAS X, Leica Microsystems, Germany). The level of baseline fluorescence was established individually for each experiment. For the visualization of drug accumulation, cells seeded on Nunc™ Lab-Tek™ chamber slides were treated with doxorubicin (0.5 μM), daunorubicin (0.5 μM), and methotrexate (5 μM) for 24 h. After washing the slides, anthracycline-treated cells were mounted with SlowFade™ glass soft-set antifade mountant (with DAPI), whereas cells incubated with methotrexate were fixed with 1% formaldehyde solution in PBS at room temperature for 15 min, and actin filaments were stained with Texas red-X phalloidin (1:1000) in 1% BSA in PBS with 0.1% TritonX-100 at room temperature for 1 h. After washing with PBS, the slides were mounted with ProLong™ diamond antifade mountant.

The induction of phosphatidylserine externalization by the anticancer drugs (24 h) in I-CBP112-pretreated cells was detected by their staining with AnnexinV-FITC. After washing with PBS, AnnexinV-FITC was added to the cells in annexin binding buffer according to the manufacturer’s instructions (room temperature, 30 min). After removing the residual unbound AnnexinV-FITC, the slides were mounted with SlowFade™ glass soft-set antifade mountant (with DAPI). Subsequently, for the imaging of the samples, the confocal laser scanning microscopy platform TCS SP8 (Leica Microsystems, Germany) with a 63×/1.40 objective (HC PL APO CS2, Leica Microsystems, Germany) was used. The samples were imaged with wavelengths of 405 and 485 nm for emission and 430–480 and 500–550 nm for excitation for DAPI and AnnexinV-FITC, respectively, using Leica Application Suite X (LAS X, Leica Microsystems). Autofluorescence-based quantification of drug accumulation

I-CBP112-treated and control cells were incubated with doxorubicin (0.5 μM), daunorubicin (0.5 μM), and methotrexate (5 μM) at 37 °C for 4 h and washed 4× with PBS, and the drug fluorescence was measured with a fluorescence microplate reader (BioTek Synergy HTX, Biokom, Poland) at the following wavelengths: 485ex/590em nm for anthracyclines and 360ex/530em nm for methotrexate. Next, the cells were lysed by freezing, and the DNA was stained with 0.1 μM DAPI at room temperature for 15 min. Fluorescence that corresponded to the DNA content was read at 360ex/460em nm. After subtracting the corresponding blanks, the autofluorescence of the drugs was normalized to the fluorescence of the DNA, and the ratio for control cells (not treated with I-CBP112) was assumed to be 1.

### 2.6. Resazurin Toxicity Assay

After incubation with I-CBP112 and drugs on Nunc^®^ MicroWell™ 384-well optical bottom plates, cells were incubated with resazurin solution (5 μM) in the growth medium at 37 °C for 4 h. The fluorescence that corresponded to the metabolic activity of living cells was measured with a fluorescence microplate reader (BioTek Synergy HTX, Biokom, Poland) at 530ex/590em nm. The fluorescence value for control cells was assumed to be 100%. The half-maximal inhibitory concentration (IC50) was calculated from the correlation binominal equation that describes the interdependence between log2 of the drug concentration and the cell viability.

### 2.7. ApoTox-Glo™ Triplex Assay

Cells were seeded and then incubated with I-CBP112 and drugs on Nunc^®^ MicroWell™ 384-well optical bottom plates. The live-cell and dead-cell protease, as well as caspase 3/7 activities, were measured with the ApoTox-Glo™ triplex assay from Promega according to the manufacturer’s instructions as described by Ling [11] with a fluorescence microplate reader (BioTek Synergy HTX, Biokom, Poland). The ratio between the chemiluminescence of caspase3/7 to fluorescent viable cells was assumed to be 1 for the control (I-CBP112- and drug-untreated) cells. Similarly, dead-cell fluorescence, which corresponds to extracellular protease activity, was normalized to the fluorescence of viable cells, which corresponds to cytoplasmic protease activity.

### 2.8. Chromatin Immunoprecipitation

Chromatin immunoprecipitation was conducted according to a previously described protocol [12]. Fragments spanning EP300 binding sites, which were detected using TFbind, at the promoters of ABCC1 and ABCC10 were amplified using KAPA SYBR^®^ FAST Universal 2× and the following primers: ABCC1 prom, 5′-ACTCAGCTTTGGAGTCAGC-3′ and 5′-CCAGGTGCAGAGAGGTTGA-3′; ABCC10 prom, 5′-CTTGTCCAAGGTCATGCAG-3′, and 5′-GCCCCACGGACAAATAATG-3′. A 10% input (sheered chromatin) was used as the internal control.

### 2.9. RNA-Seq Analysis in Galaxy Version 19.05.dev

The following data from MDA-MB-231 cells were used for the RNA-Seq analysis: siCTRL, GSM2736169 (SRR5919378), and GSM2736170 (SRR5919379); siBRDa, GSM2736175 (SRR5919384), and GSM2736176 (SRR5919385); siBRDb, GSM2736177 (SRR5919386), and GSM2736178 (SRR5919387); DMSO, GSM2736179 (SRR5919388) and GSM2736180 (SRR5919389); and JQ1, GSM2736181 (SRR5919390), and GSM2736182 (SRR5919391) [13].

The data in FASTQ format were unified to Sanger formatting with FASTQ Groomer and then mapped to Human Genome version 19 using TopHat [14,15]. Transcripts were assembled with Cufflinks (using UCSC Known Gene as a reference annotation) and merged using Cuffmerge [16]. Differential gene expression was determined with CuffDiff using UCSC on Human:gtexGene (hg19_gtexGene) as a template. The numbers of cDNA fragments for the chosen genes are reported as fragments per kilobase of transcript per million mapped reads (FPKM).

### 2.10. Statistical Analysis

The data in Table S1 are reported as the mean ± standard deviation of the mean (SEM). The Student’s *t*-test was used to determine statistically significant differences between two means (marked with * when *p* < 0.05, ** when *p* < 0.01, and *** when *p* < 0.001), whereas one-way or two-way analysis of variance (ANOVA1 or ANOVA2, respectively) was conducted in GraphPad Prism 5 to compare means across several groups. ANOVA1 was followed by the Tukey post hoc test, and ANOVA2, by Bonferroni tests. Statistically significant differences are marked with * when *p* < 0.05, ** when *p* < 0.01, and *** when *p* < 0.001.

## 3. Results

### 3.1. I-CBP112 Augmented the Toxicity of Anticancer Drugs

**Figure 1:**
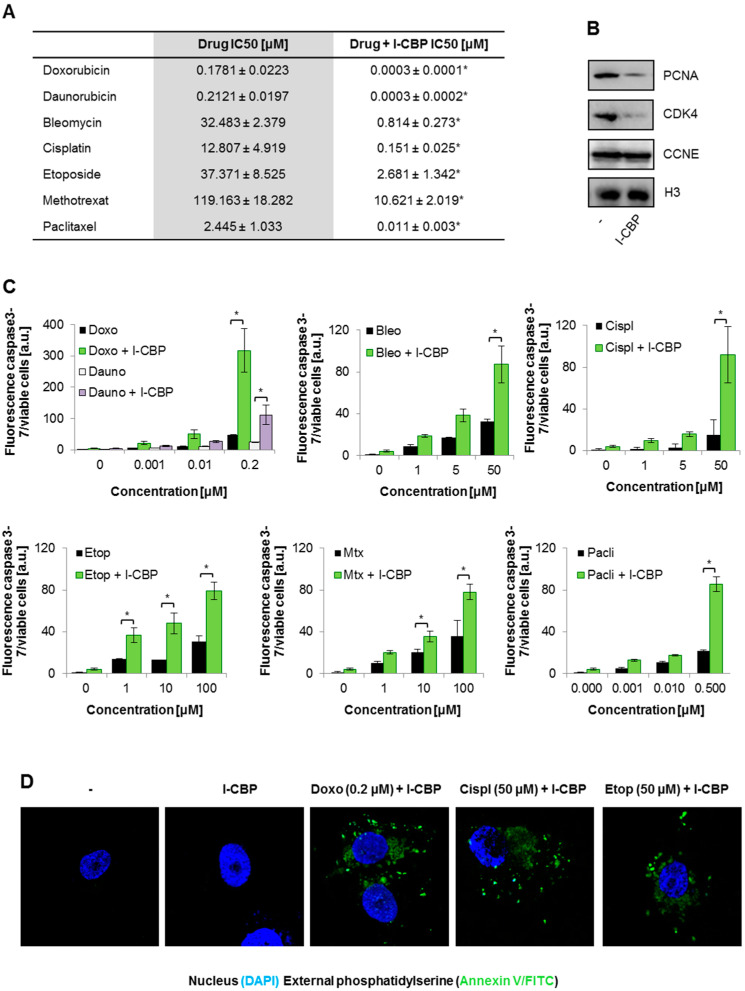
I-CBP inhibited proliferation and sensitized MDA-MB-231 breast cancer cells to anticancer drugs. (**A**) The half-maximal inhibitory concentrations (IC50s) for the seven studied drugs were calculated from the results of the resazurin-based viability assay. I-CBP112 (10 µM) was added to cells for 72 h prior to treatment with chemotherapeutics, which were added for 48 h. The IC50 was determined using the binominal equation. (**B**) The effect of 72 h cell incubation with I-CBP112 on proliferation was studied by visualizing the protein levels of PCNA, CDK4, and CCNE in the compound-treated and untreated cells by Western blotting. H3 was used as the loading control. (**C**) The ratio between caspase-3/7 cleaved substrate and live-cell protease activity was quantified to estimate the modulatory potential of I-CBP112 (10 µM; 72 h) on drug-induced apoptosis. Features of apoptosis, necrosis, and living cells were monitored 24 h after their treatment with drugs. (**D**) Externalization of phosphatidylserine, which marks apoptotic cells, was monitored by confocal microscopy after cell staining with FITC-conjugated annexinV (green). DNA was stained with DAPI (blue). The scheme of cell treatment was the same as in (**C**). All data in bars are reported as mean ± SEM. (**A**) The difference between two means was tested with Student’s *t*-test, and statistically significant differences are marked with * when *p* < 0.05. (**C**) The impact of drug-induced apoptosis was tested with two-way ANOVA and the Bonferroni test. Statistically significant effects of I-CBP112 are marked with * when *p* < 0.05.

First, to test the possible influence of I-CBP112 on the breast cancer cells’ response to drugs, we pretreated MDA-MB-231 cells with 10 μM bromodomain inhibitor for 48 h and tested the toxicity of selected anticancer chemotherapeutics across wide ranges of concentrations using the resazurin assay. As shown in Figure 1A and Table S1, doxorubicin and daunorubicin showed the highest and similar (IC50—0.178 and 0.212, respectively) cytotoxicity in the culture of breast cancer cells. Antimicrotubule agent paclitaxel was approximately 100× less effective than the alkylating agent cisplatin 600× when compared to anthracyclines. Glycopeptide antibiotic (bleomacin) and topoisomerase 2 inhibitor (etoposide) and similarly reduced the number of living MDA-MB-231 cells to 50% at the concentration of 32.5 and 37.4 μM, respectively. Among all the considered drugs, methotrexate turned out as the least cytotoxic with IC50 ~119 μM.

I-CBP112 considerably decreased the IC50 of all of the studied drugs from 10 (methotrexate and etoposide) to over 600-fold (anthracyclines). The concentration of bleomycin, which was required to reduce by half the number of metabolically active breast cancer cells in the presence of I-CBP112, decreased ~40-fold, concentration of cisplatin declined ~85-fold, whereas paclitaxel ~222-fold. I-CBP112 alone reduced the number of viable cells in the culture to approximately 60%. This effect seemed to be caused by the disturbance of cell cycle progression, since the analysis of mitotic division markers (Figure 1B and Figure S1) indicated reduced levels of PCNA and CDK4 but not cyclin E. This conclusion is further supported by the data in Figure 1C, Figure S3A and Table S1, showing that I-CBP112 alone did not trigger the activation of caspase 3/7 activity, neither increased the activity of dead-cell protease in the cell culture media.

All of the studied drugs caused a relatively strong increase in caspase 3/7 activity at the highest concentrations (Figure 1C, Table S1) and only slight, but statistically significant, enhancement in extracellular protease activity (Figure S3A, Table S1; ANOVA2—drug concentration affected variance of the group means). CBP/EP300 inhibitor intensified caspase 3/7 activity mostly when combined with the highest tested doses of all the anticancer drugs (Figure 1C), but increased cell membrane permeability only when added prior to 0.2 μM daunorubicin. Notably, when mixed with etoposide, methotrexate, and a low concentration of cisplatin, I-CBP112 decreased the ratio between dead-cell and live-cell fluorescence.

Cells treated with the combination of I-CBP112 and drugs were also stained with annexinV-FITC (Figure 1D and Figure S2). The addition of doxorubicin (0.2 μM), cisplatin (50 μM), and etoposide (50 μM) to I-CBP112-pretreated cells caused visible externalization of phosphatidylserine without visible changes in the nucleus structure (a lack of chromatin condensation or fragmentation). Importantly, the bromodomain inhibitor alone did not induce the exposure of phosphatidylserine on the outer plasma membrane, providing further evidence for a lack of direct I-CBP112 toxicity for MDA-MB-231 cancer cells.

This all may suggest that the considered epigenetic inhibitor primes MDA-MB-231 cells for apoptotic cell death in response to anticancer drugs. The selected time point (48 h after drug administration in I-CBP112) for the cell death readout captures cells in the early apoptosis.

### 3.2. CBP/EP300 Inhibitor Enhanced Drug Accumulation and Phenocopied the Effect of Pan-ABC Inhibitor on Drug Toxicity

**Figure 2:**
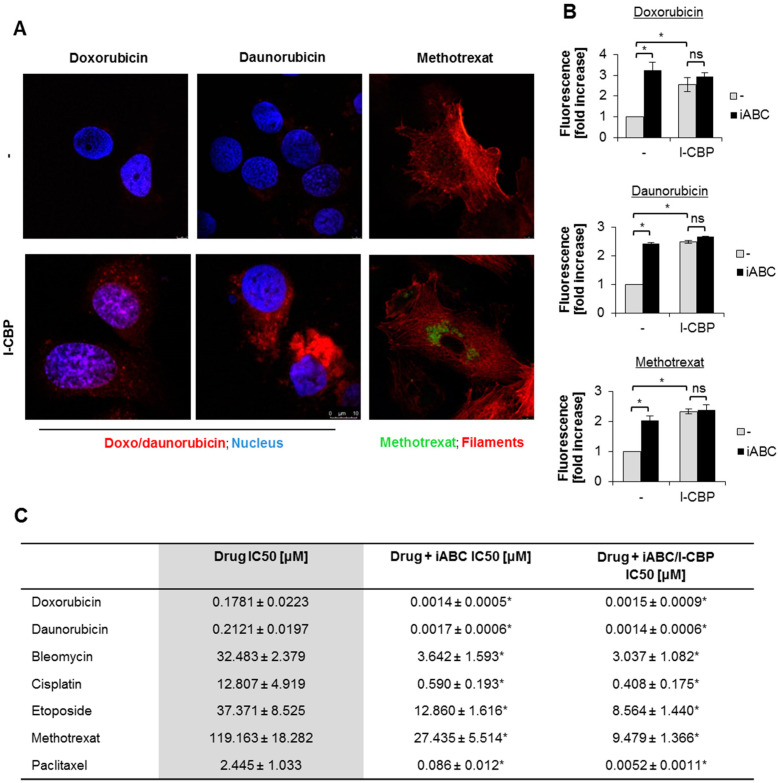
I-CBP increased the accumulation of some anticancer drugs and phenocopied the effect of ABC inhibitor in MDA-MB-231 breast cancer cells. (**A**) A confocal microscope was used to track doxorubicin, daunorubicin, and methotrexate autofluorescence in MDA-MB-231. The nuclei in anthracycline (red)-treated cells were additionally stained with DAPI (blue), whereas actin filaments were stained with phalloidin-TexasRed (red) conjugate in cells incubated with methotrexate (green). To cells, we added anthracyclines (0.5 µM) and methotrexate (5 µM) for 24 h. (**B**) The autofluorescence of cells incubated with the drugs for 4 h was measured using a fluorescence reader. iABC (probenecid; 200 µM) was added 2 h prior to cell treatment with anticancer drugs. (**C**) IC50 for anticancer drug toxicity was estimated using the resazurin viability assay. iABC (probenecid; 200 µM) was added 2 h, whereas I-CBP112 (10 µM) was added 72 h, prior to cell treatment with anticancer drugs. Bars in the figures represent the mean ± standard error of the mean (SEM). (**B**) Variability among groups was tested with two-way ANOVA and the Bonferroni test. Statistically significant effects of I-CBP112 are marked with * when *p* < 0.05. (**C**) Two-way ANOVA and the Bonferroni test were used to test variance among the IC50 values of control, I-CBP112-, iABC-, and iABC/I-CBP112-pretreated cells. The effect of I-CBP112 is marked with * when *p* < 0.05.

As I-CBP112 increased anticancer drugs’ toxicity and might have controlled the transcription of some multidrug-resistance-relevant genes that belong to the family of ABC transporters, we tested if the bromodomain inhibitor affected the intracellular levels of some drugs. We used the autofluorescence of two anthracyclines, which also showed the most striking decreases in IC50 when combined with I-CBP112, and methotrexate for their visualization by confocal microscopy. As shown in Figure 2A and Figure S2, cell incubation with I-CBP112 for 48 h substantially increased the distribution of drugs in the nuclei (doxorubicin) and cytoplasm (daunorubicin and methotrexate). The quantification of the drug levels inside cells confirmed higher levels of chemotherapeutics in the cells pretreated with I-CBP112 (Figure 2B, Table S1). Importantly, the CBP/EP300 inhibitor did not act synergistically with the pan-ABC inhibitor probenecid, whereas the individual impact of each of the two compounds on the drug accumulation was comparable. The Bonferroni post hoc test indicates a statistically significant effect of I-CBP112 on the drug accumulation only in iABC-untreated cells.

Since I-CBP112 and iABC allowed for an increased drug level inside the cells but did not strengthen the action of each other, we examined if similar interdependence could be observed in the degree of cell sensitization to anticancer drugs. Based on the resazurin toxicity assay, we estimated the IC50s for particular drugs in cells pretreated with iABC alone and in combination with I-CBP112, and compared the calculated values for the control and I-CBP112-pretreated cells. As shown in Figure 2C and Table S1, probenecid augmented the drug toxicity similarly to the CBP/EP300 inhibitor. Statistical analysis with two-way ANOVA and the Bonferroni test again indicated that the effect of I-CBP112 on cell viability was only observed in the absence of iABC. These results suggest that both compounds act in the same regulatory circuit and, in this particular case, modulate cell vulnerability to chemotherapeutics by inhibiting drug efflux.

### 3.3. Expression of ABC Transporters Is Downregulated by I-CBP112 in MDA-MB-231

**Figure 3:**
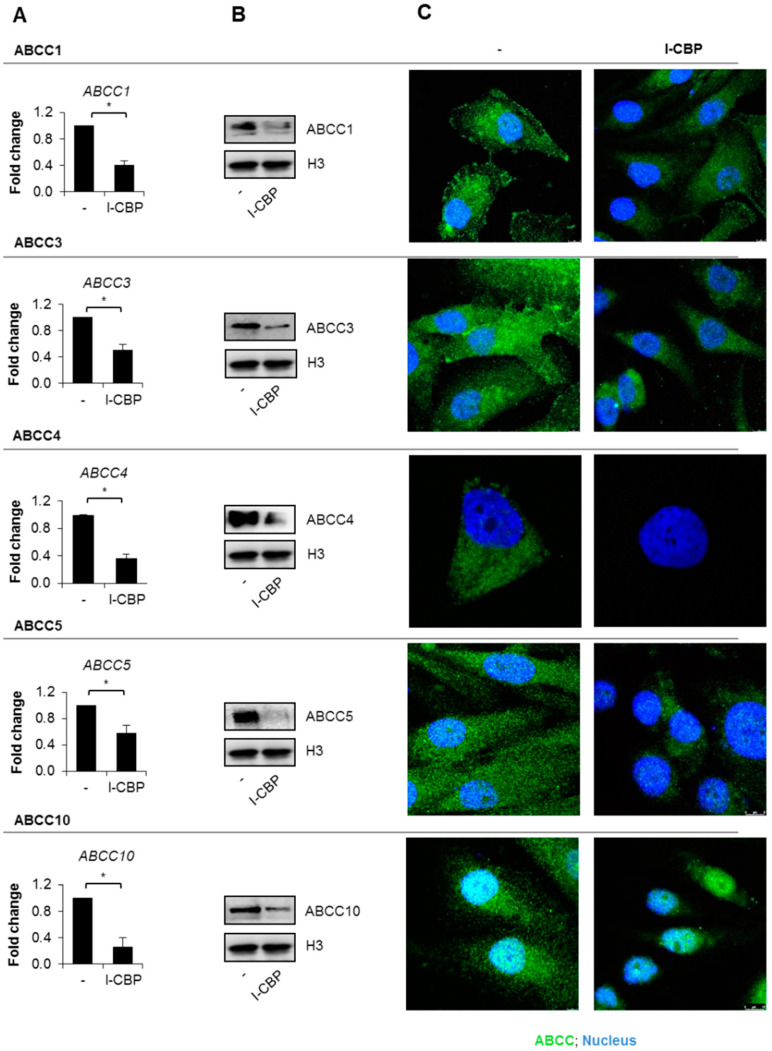
I-CBP 112 decreased the expression of ABCC1, ABCC3, ABCC4, ABCC5, and ABCC10 in the MDA-MB-231 cell line. (**A**–**C**) Cells were incubated with 10 µM I-CBP112 for 72 h. (**A**) The mRNA levels of selected ABC transporters compared between the control and I-CBP112-treated cells by real-time PCR. ABC gene expression was normalized to the mRNA levels of ACTB and GAPDH. (**B**) The same transporters were visualized in cell lysates by Western blotting, and H3 was used as a loading control. (**C**) ABC protein level and localization were compared through confocal microscopy using AlexaFluor488-conjugated secondary antibody (green). DNA was stained with DAPI (blue). (**A**) Bars represent mean ± standard error of the mean (SEM). The differences between 2 means were tested with Student’s *t*-test and are marked with * when *p* < 0.05.

Knowing that I-CBP112 allowed for a higher drug concentration in the breast cancer cell line and phenocopied iABC in terms of drug accumulation and cell sensitization to anticancer therapeutics, and that I-CBP112 altered nucleosome acetylation, we verified if the CBP/EP300 inhibitor affected the transcription and protein levels of ABC proteins, which, according to the literature, contribute to multidrug resistance. From the list of MDR1-7, we chose those that were the most abundant in MDA-MB-231. As shown in Figure 3A, cell incubation with I-CBP112 for 72 h led to considerable repression of ABCC1, ABCC3, ABCC4, ABCC5, and ABCC10.

The decrease in mRNA levels was followed by a reduction in ABCC1/3/4/5/10 proteins (Figure 2B,C, Table S1). ABCC3 and ABCC4 were mostly observed in the cytoplasm, and ABCC10, in the nucleoplasm, whereas ABCC1 and ABCC5 were equally distributed across the cell (Figure 3C, Figure S2). I-CBP112 did not considerably affect the cellular localization of ABC proteins but caused a visible decrease in the green fluorescence that corresponded to the transporter protein level. The quantification of the green fluorescence intensity in the control and I-CBP112-treated cells is provided in Figure S3B and Table S1.

### 3.4. The Drug Resistance was also Decreased by I-CBP112 in Other Cell Lines

**Figure 4:**
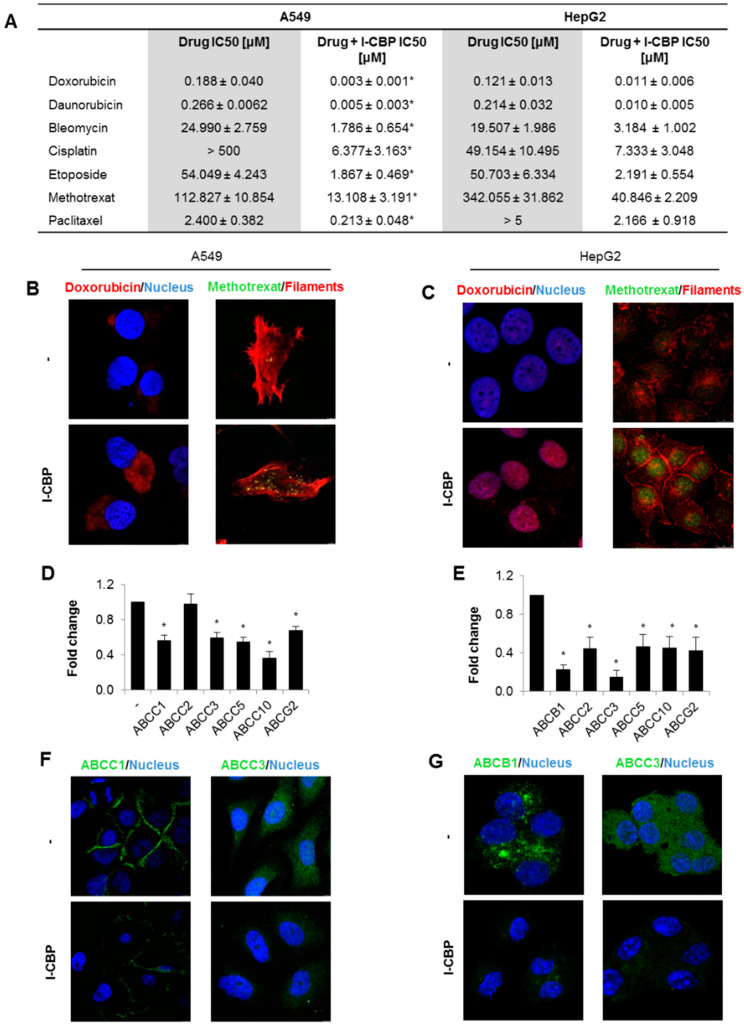
I-CBP112 sensitized other cancer cell types to chemotherapeutics, increased drug accumulation in cells, and reduced expression of ABC transporters. (**A**) IC50s were evaluated in another two cell lines—A549 lung cells and HepG2 hepatocytes—with the resazurin toxicity assay. Cells were incubated with 10 µM I-CBP112 for 72 h prior to the administration of drugs for another 48 h. IC50 was determined from the binominal equation (Supplementary Statistics: resazurin A549 and resazurin HepG2). The autofluorescence of doxorubicin and methotrexate was imaged in control and I-CBP112 (10 µM; 72 h)-pretreated A549 (**B**) and HepG2 (**C**) cells using a confocal microscope. Doxorubicin-treated cells (0.5 µM; 24 h) were additionally stained with DAPI (blue, nucleus), whereas methotrexate-treated (5 µM; 24 h) cells were additionally stained with phalloidin-Texas Red (red, actin filaments). (**D**,**E**) The impact of I-CBP112 (10 µM; 72 h) on ABC gene expression was measured by real-time PCR in A549 (**D**) and HepG2 (**E**) cell lines. The mRNA levels of particular genes were normalized to ACTB and GAPDH and assumed to be 1 for control cells. (**F**,**G**) Example of confocal images of ABC proteins that are highly abundant in A549 (**F**) and HepG2 (**G**) cells. I-CBP112 (10 µM) was added for 72 h. Transporters were stained in green (secondary anti-rabbit AlexaFluor488-conjugated antibody), and nuclei, in blue (DAPI). (**A**,**D**,**E**) bars represent the mean ± standard error of the mean (SEM). (**A**) IC50 values for the control and I-CBP112-treated cells were compared using Student’s *t*-test, and statistically significant differences between means are marked with * when *p* < 0.05. The same analysis was used to compare relative mRNA levels in (**D**,**E**).

To test the cancer-cell-type specificity of I-CBP112’s effects on the toxicity of drugs and their accumulation inside cells, we repeated the evaluation of certain drug-resistance parameters in two other cell lines: the non-small cell lung epithelial cancer cell line A549 and hepatocyte carcinoma line HepG2. These two cell lines differ in their profiles of MDR and glycoprotein-P transcription according to The Human Protein Atlas. The two cancer cell lines substantially differed from MDA-MB-231 and from each other in the vulnerability to anticancer drugs (Figure 4A, Table S1). Invariably, anthracyclines emerged most toxic among the considered chemotherapeutics (IC50 < 0.28 μM) in A549 and HepG2 cells. Doxorubicin, daunorubicin, etoposide, and bleomycin showed similar cytotoxicity in the two cell lines, but their response to methotrexate, paclitaxel and, particularly, to cisplatin varied.

The preincubation of both cell lines with I-CBP112 for 48 h considerably affected IC50 values for all the tested chemotherapeutics from 8.6-fold to 78.2-fold in the culture of A549, but only from 2.3-fold to 23.1-fold in HepG2. CBP/EP300 inhibitor potentiated most the action of cisplatin (78.2-fold decline in IC50), then doxorubicin (62.7-fold), daunorubicin (53.2-fold), etoposide (28.9-fold), bleomycin (14-fold), paclitaxel (11.3-fold) and methotrexat in lung cancer cells. In hepatocytes the impact of I-CBP112 on the drug toxicity declined as follows: etoposide (23.1-fold decrease in the IC50 value), daunorubicin (21.4-fold), doxorubicin (11-fold), methotrexate (8.4-fold), cisplatin (6.7-fold), bleomycin (6.1-fold), and paclitaxel (2.3-fold).

In these cancer cell lines, higher concentrations of doxorubicin and methotrexate were observed in cells preincubated with I-CBP112 (Figure 4B,C and Figure S2). In A549, the drugs were mostly localized in the cytoplasm, whereas in HepG2, doxorubicin, and methotrexate they were mostly enriched in the nuclei. A549 cells responded to the CBP/EP300 inhibitor, with the repression of ABCC1, ABCC3, ABCC5, ABCC10, and ABCG2, but not ABCC2, whereas all the considered ABCC and ABCG2 transporters were found to be decreased in HepG2 (Figure 4D,E, Table S1). The latter cell line was also characterized by a decline in ABCB1 transcription. The I-CBP112-induced transcriptional inhibition of ABC transporters was followed by a visible decline in their protein levels (exemplary confocal images of selected transporters are shown in Figure 4F,G and Figure S2). In summary, I-CBP112 considerably inhibits the expression of genes functionally linked to drug efflux regardless of the cancer cell origin, and decreases the IC50s of all the considered anticancer therapeutics, which vary in their mechanisms of cancer killing.

### 3.5. CBP/EP300 Inhibitor Induced Recruitment of LSD1 to the Promoters of ABCC1 and ABCC10 and Erased Trimethylation of H3K4 in MDA-MB-231

**Figure 5:**
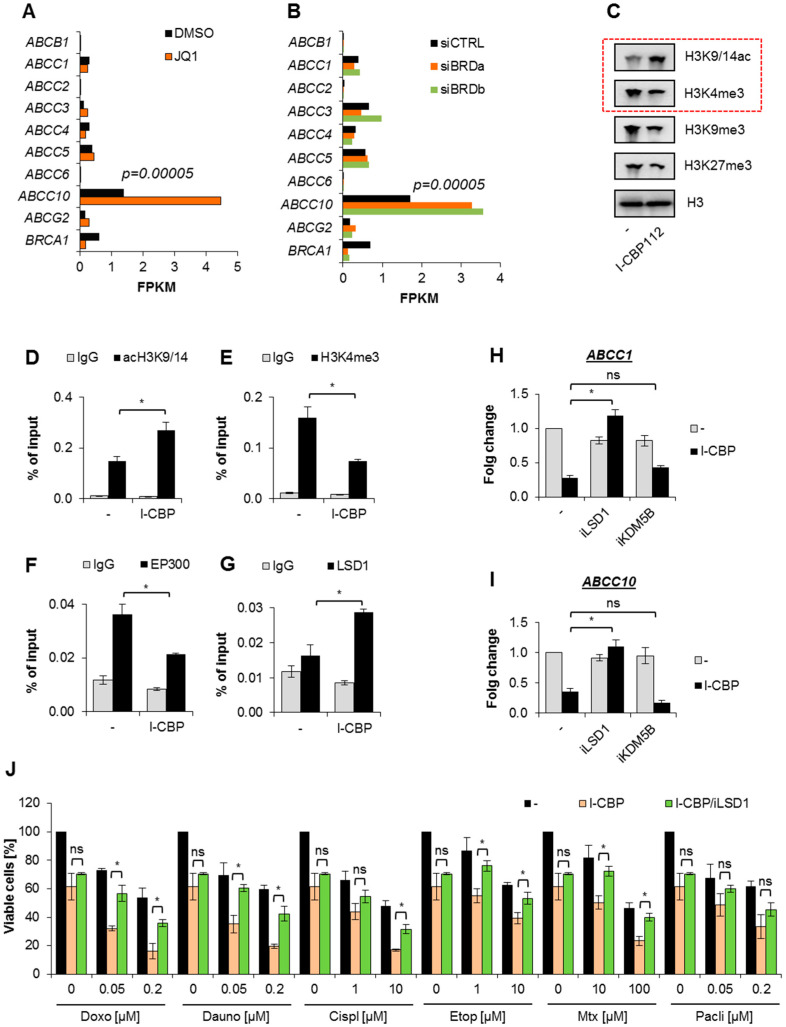
I-CBP112 reduced trimethylation of H3K4 and induced LSD1-mediated gene repression in MDA-MB-231 breast cancer cells. (**A**,**B**) The impact of BRD silencing and JQ1 (bromodomain inhibitor) on the mRNA levels of selected ABC transporters was determined by quantifying differential gene expression (using TopHat for mapping, CuffLinks for transcript assembly, and CuffDiff for quantitative transcript comparison) based on publicly available data sets. Gene expression is presented as fragments per kilobase of transcript per million mapped reads (FPKM). (**C**) The effect of I-CBP112 (10 µM; 72 h) on some histone modifications was evaluated in cell lysates by Western blotting. Transcription-promoting markers are highlighted using red dashed rectangles. (**D**–**G**) Alterations in two histone modifications and in the occurrence of EP300 and LSD1 caused by I-CBP112 (10 µM; 72 h) at the promoter of ABCC10 were assayed by ChIP-qPCR. (**H**,**I**) The impact of iLSD1 (SP2509; 0.1 µM) and iKDM5B (PBIT; 2.5 µM) on I-CBP112-induced ABCC1 and ABCC10 gene repression was estimated using real-time PCR. iLSD1 and iKDM5B were added alone or in combination with I-CBP112 (10 µM) for 72 h. (**J**) The resazurin viability assay was used to test the effect of iLSD1 on the I-CBP112-induced increase in cell vulnerability to anticancer drugs. iLSD1 (0.1 µM) was added to cells in combination with I-CBP112 (10 µM) for 72 h, and the cells were then treated with two selected doses of chemotherapeutics for another 48 h. Bars in the graph show the relevant cell viability normalized to the untreated control, which was assumed to be 100%. Bars in the figures represent the mean ± standard error of the mean (SEM). (**D**–**G**) Means were compared using Student’s *t*-test, and statistically significant differences are marked with * when *p* < 0.05. (**H**,**I**) The influence of particular factors on gene transcription was analyzed by two-way ANOVA and the Bonferroni test, and statistically significant differences in the variance are marked with * when *p* < 0.05. (**J**) Data were tested with one-way ANOVA and Tukey’s post hoc test. The significant impact of iLSD1 on I-CBP112-induced increase in drug toxicity is marked with * when *p* < 0.05.

In search for the molecular mechanism responsible for the observed repression of multidrug-resistance proteins and glycoprotein-P, we first considered the possible impact of I-CBP112 bromodomain inhibitor on the function of bromodomain and extraterminal domain (BET; BRD2, BRD3, BRD4, and BRDT) family of bromodomain proteins in transcription control of the key multidrug resistance genes. BET proteins, as other bromodomain-containing proteins, interact with acetylated histones and transcription factors, hence are involved in transcriptional regulation. Therefore, we assumed that BET proteins may act as activators of some ABCs’ transcription and I-CBP112 may interfere with their functional interaction with the gene promoters. We used publicly available RNA-Seq data to test the impact of the known BET inhibitor JQ1 and transient silencing of bromodomains (2-3-4) on the mRNA levels of ABC transporters in MDA-MB-231. As shown in Figure 5A, JQ1 significantly enhanced the transcription of ABCC10 but only slightly altered the mRNAs of other genes. A similar profile of changes was observed in breast cancer cells transfected with siBRD, where only the highly transcribed ABCC10 responded to BRD (2-3-4) deficiency, with further transcription enhancement (Figure 5B). BRCA1 is shown as an example of gene inhibited upon deficiency of some BET members (Figure 5B) and their activity (Figure 5A). Since the inhibition and silencing of some BET proteins resulted in the upregulation of one ABC gene, the observed repression of most of the considered ABC transporters by I-CBP112 in three different cell lines seemed to be rather mediated by a BET-independent mechanism.

As I-CBP112 was documented as capable of inhibiting CBP/EP300 interaction with chromatin and of simultaneously increasing nucleosome acetylation, we compared the extent of H3K9/14 acetylation between the control and I-CBP112-treated MDA-MB-231 cells. In addition, we also examined some other histone modifications that contribute to transcriptional regulation. The Western blot images in Figure 5C and Figure S1 indicate the strong enrichment in H3K9/14ac that was simultaneously associated with a decline in histone methylation status. If the losses of H3K9me3 and H3K27me3 occurred along with increased H3K9/14 acetylation, which suggested that I-CBP112 favored a transcription-permissive epigenetic status, the decline in H3K4me3 was generally linked to the gene’s repression. In the next step, we tested the acetylation and H3K4me3 statuses at the promoters of the highly transcribed ABCC10 and the key MDR gene—ABCC1. The alteration of the considered transcription-promoting epigenetic markers at the gene promoters phenocopied the I-CBP112-induced modifications observed with Western blotting; increased histone acetylation was followed by a decline in H3K4me3 (Figure 5D,E, Figure S3C, Table S1). Despite enhanced nucleosome acetylation, some extrusion of EP300 and BRG1/SMARCA4 from the gene promoter was also noted, which may indicate the impact of I-CBP112 on the interaction between bromodomain-containing EP300 and DNA (Figure 5F, Table S1). The decrease in the trimethylation of H3K4 at the promoters of ABCC10 and ABCC1 prompted us to check for the occurrence of lysine-specific demethylase 1A (LSD1), which demethylates mono- and di-methylated lysines, specifically histone 3, and lysines 4 and 9 (Figure 5G, Figure 3B, Table S1). The incubation of breast cancer cells with I-CBP112 triggered a statistically significant enrichment in LSD1 at the two gene promoters.

To verify the possible contribution of LSD1 and/or KDM5B, which removes methyl groups from trimethylated, demethylated, and monomethylated H3K4, we treated MDA-MB-231 cells with the combination of I-CBP112 and iLSD1 (0.1 µM SP2901) or I-CBP112 and iKDM5B (5 µM PBIT). As shown in Figure 5H,I, and Table S1, iLSD1 prevented the I-CBP112-induced repression of ABCC1 and ABCC10, whereas iKDM5B did not interfere with the studied genes’ responses to the CBP/EP300 inhibitor. LSD1 silencing with siRNA resulted in the massive death of MDA-MB-231 (data not shown); therefore, further validation of the possible impact of I-CBP112-LSD1 crosstalk on gene transcription in an LSD1-deficient background was impossible. Regardless, the observed recruitment of demethylase to the gene promoters and the antagonizing effect of iLSD1 with I-CBP112 on the gene transcription suggest that I-CBP112 may repress ABC genes’ transcription by triggering the enrichment of LSD1 at their promoters.

As the CBP/EP300 inhibitor reduced the viability of drug-treated cells and this effect might have occurred due to the repression of multidrug-resistance proteins, we expected that iLSD1 might, at least partially, counteract I-CBP112-enhanced drug toxicity. To verify this hypothesis, we compared the viability of MDA-MB-231 cells exposed to two concentrations of anticancer drugs, which were pretreated with a combination of I-CBP112 (10 µM) and iLSD1 (0.1 µM), to that of those treated with I-CBP112 (10 µM) alone. iLSD1 considerably suppressed I-CBP112’s potential to enhance the toxicity of all the studied compounds, except for paclitaxel, at the highest tested concentrations and rescued the I-CBP112-induced decline in cell viability (Figure 5J, Table S1). These findings all suggest that I-CBP112 may considerably weaken cancer cells’ resistance to chemotherapeutics by repressing the transcription of key ABC transporters in an LSD1-dependent manner.

## 4. Discussion

Inhibitors of bromodomain extra-terminal (BET) and non-BET families appear to be promising anticancer drugs due to their cellular function and deregulation of their target proteins in different tumor types. Although several BRD inhibitors have entered clinical trials over the last decade, they have experienced significant obstacles such as a lack of partial or complete response and high efficacy in only a few specific tumor types (hematological malignancies and rare diseases such as NUT) [17]. These features have precluded their regulatory approval. For triple-negative breast cancer, the study registered under NCT02698176 aimed to target advanced solid tumors with birabresib (MK-8628) in monotherapy, but one recruited participant with TNBC did not finish the treatment because of progressive disease, and the entire trial was terminated due to the limited efficacy in other tumor types (not due to safety reasons) [18]. Therefore, no conclusion can be drawn regarding the anticancer activity of bromodomain inhibitors in patients diagnosed with TNBC. In the in vitro and in vivo MDA-MB-231 murine xenograft models, the above-mentioned birabresib showed antiproliferative activity, repressed the c-MYC protein, and synergized with the mTOR inhibitor everolimus [19]. We observed a similar effect of I-CBP112, which targets the CBP/EP300 bromodomain, on the mitotic division in a culture of MDA-MB-231, whereas a substantial reduction in c-MYC, which is considered a TNBC driver, was observed in prostate cancer [9]. Nothing is known about the in vivo activity of I-CBP112. Since the clinical trial with birabresib (MK-8628) did not report relatively high toxicity, I-CBP112 may be similarly or better tolerated, particularly when the toxicity profiles of BET inhibitors, which include thrombocytopenia, fatigue, and diarrhea, emerge as class effects [17].

I-CBP112, similar to birabresib and other bromodomain inhibitors, synergistically reduces the numbers of viable cells in culture when added to cultures with other anticancer drugs. This approach, administering a bromodomain inhibitor in combination with various oncology therapeutics, was also tested in clinical trials. In an active, non-recruiting phase 2 study on patients with TNBC without germline mutations of BRCA1 or BRCA2, ZEN003694 was mixed with the PARP inhibitor talazoparib, and the primary completion of the study is estimated to occur in September 2021 (NCT03901469). The safety and tolerability study of the treatment scheme comprising INCB057643 and gemcitabine, paclitaxel, rucaparib, abiraterone, ruxolitinib, or azacitidine in subjects with advanced malignancies, including breast cancers, was terminated due to safety issues (NCT02711137). When considering the joint treatment of tumors with bromodomain inhibitors and other anticancer drugs, the possible impact of the first group of compounds must be taken into account. As shown in Figure 4A,B, the deficiency in bromodomain activity may lead to the overexpression of ABC proteins, some of which are responsible for the active removal of anticancer therapeutics. In the studied MDA-MB-231 breast cancer cells, the transient silencing of BRD2/3/4 and bromodomain inhibition caused a significant increase in the mRNA of ABCC10, which is the most abundant MDR protein in this cell line. Importantly, JQ1 binds to all the bromodomains of the BET family but not to bromodomains outside the BET family. This suggests that BET family members act as anti-MDR factors, at least in the studied triple-negative breast cancer cell line. The possible issues with the transcriptional upregulation of ABC transporters may be precluded by replacing BET inhibitors with I-CBP112. The latter compound phenocopies the anticancer activity of BET inhibitors in in vitro models and considerably augments the accumulation of anticancer drugs by repressing genes responsible for multidrug resistance. I-CBP112 seems to be specific for CBP/EP300, because no activity of this compound was observed for BRG1/SMARCA4 occurrence on the promoter of ABCC1 (Supplementary Table S1). Our results suggest that I-CBP112 may increase the toxicity of these therapeutics, which are actively removed from cells by ABC transporters. The anti-MDR activity of I-CBP112 seems to be cancer-type independent because the expression of ABC genes was repressed in the breast, lung, and hepatic cell lines. Moreover, this bromodomain inhibitor was potent in reducing the mRNA levels of most of the examined transporters. The aspects that must be further evaluated concern I-CBP112’s toxicity and the optimal treatment scheme, which includes the time between I-CBP112 and anticancer drug administration. Since the CBP/EP300 inhibitor represses gene transcription, the expected impact on drug accumulation is delayed. The pretreatment of our three cell lines grown in a monolayer with I-CBP112 for 72 h was sufficient to substantially reduce the ABC protein levels in the cell membranes, but the variability in the exposure of the tumor cells to the drugs may alter their expected responses to I-CBP112.

The described approach to decreasing multidrug resistance and ABC transporter expression by targeting chromatin-interacting enzyme(s) is new and paves the way for the repression of drug-efflux-associated membrane proteins at the genomic level. Previous and current attempts to overcome native and acquired drug resistance have focused on the development of inhibitors of ABC proteins, mainly P-glycoprotein (ABCB1), BCRP (ABCG2), and MRP1 (ABCC1); some of these compounds show weak specificity and show the potential to inhibit more than one ABC transporter [20,21]. Some papers also describe the possible involvement of flavonoid and terpenoid derivatives as ABC transporter modulators [22]. However, none of the proposed ABC inhibitors were approved for anticancer therapy. A total of 98 small molecules that possess P-glycoprotein-inhibiting properties were approved by the FDA, with indications for non-cancer diseases [23]. Novel strategies to limit or reverse multidrug resistance include miRNA, DNA methyltransferase inhibitors (DNMTis), hypomethylating agents (HMAs), and histone deacetylase inhibitors (HDACis) [24], but HDAC inhibitors elicit divergent responses in drug-sensitive and resistant cancer cells [25]. HDAC inhibitors are usually associated with the activation of gene transcription because they prevent histone deacetylation, but numerous other HDAC non-histone HDAC targets have different impacts on gene transcription depending on their acetylation statuses. As for histones, their hyperacetylation induced by I-CBP112 was associated with decreased H3K4me3 and the recruitment of LSD1 at the promoters of ABCC1 and ABCC10 (Figure 5D,E,G and Supplementary Table S1). Since the mechanism responsible for LSD1 enrichment at the hyperacetylated promoters and the superior role of H3K4me3 declines with increased acetylation in transcription, the efficacy remains unknown, so further experiments are needed to test the possible impact of HDAC inhibitors on the transcription of genes functionally involved in multidrug resistance. There have been considerable advances in the study of cancer cells that acquire resistance as a consequence of the repeated administration of anticancer drugs because such cells are frequently characterized by the overexpression of ABC proteins, which are crucial for drug efflux. Further questions to be answered include the impact of I-CBP112 on ABC transcription and promoter acetylation, and whether I-CBP112 causes the hyperacetylation of the promoters of overexpressed genes and LSD1 recruitment. Such a model may help in finding links or discrepancies between simultaneous adverse shifts in transcription-promoting histone markers and in verifying the role of LSD1.

The recruitment of LSD1 to the promoters of highly transcribed genes such as ABCC10 is unexpected, particularly when the acetylation of the ABCC10 promoter is further enhanced by I-CBP112. LSD1 lacks a bromodomain, and the known crystal structure of the enzyme does not indicate any protein fragment with an acetylated-histone-reader function. Conversely, LSD1 is most frequently associated with the CoREST repressor complex, which comprises HDAC1 and HDAC2; hence, LSD1 activity and the demethylation of H3K4me3 are associated with histone deacetylation. LSD1 binds DNA and anchors associated proteins or protein complexes to nucleosomal substrates via the evolutionarily conserved SWIRM domain [26,27], which closely interacts with the amine oxidase domain, forming a highly conserved cleft, and may, therefore, serve as an additional histone-tail-binding site. The SWIRM domain of human ADA2alpha was shown to colocalize with lysine-acetylated histone H3 in the cell nucleus [28]. Moreover, SWIRM-containing ADA2b is required for the efficient acetylation of histone tails by GCN5 [29]. However, nothing is known about the interaction between LSD1 and acetylated nucleosomes. A relatively recent paper describes the interdependence between the acetylation of particular amino acids in histone H3 and the deacetylase and demethylase activities of the epigenetic silencing complex CoREST [30]. The demethylase activity of methyl-Lys4 in histone H3 was strongly inhibited by H3 Lys14 acetylation, but the presence of this modification at H3 Lys18 considerably increased the Km for LSD1-catalyzed histone demethylation. We know nothing about the selectivity of CBP/EP300 histone acetylation at particular histone residues or about its possible impact on LSD1 and CoREST’s repressive activity. Another aspect that needs to be mentioned is the contribution of another demethylase in I-CBP112-induced gene repression: LSD1 can only demethylate mono- or di-methylated lysine residues on histone H3 because the trimethyl-lysine residue is not protonated [31]. This suggests that the activity of LSD1 is preceded by that of another enzyme that removes the first methyl group from trimethylated H3K4. None of the bromodomain-containing proteins are capable of the above-mentioned function [32]. However, LSD1 is as a key repressor of multidrug-resistant proteins in I-CBP112-treated MDA-MB-231 cells, since LSD1 inhibition prevents the I-CBP112-induced decline in ABCC1 and ABCC10 transcription. Since some of LSD1 inhibitors are tested in clinical trials and emerge as promising agents for anticancer approaches, the attention should be paid to their possible pro-multidrug resistance action under certain circumstances.

## 5. Conclusions

In summary, I-CBP112 has emerged as a promising compound for reducing the innate drug resistance in the studied cancer cells. The studied compound represses expression of ABCC1, ABCC3, ABCC4, ABCC5, and ABCC10, and increases the accumulation of some anticancer drugs. At the gene promoters of ABCC1 and ABCC10, I-CBP112 causes chromatin compaction and the removal of transcription promoting trimethylation of H3K4 by LSD1, but simultaneously intensifies nucleosome acetylation. The molecular interdependence between these two chromatin features, as well as the mechanism that allows for LSD1 recruitment to hyperacetylated promoters, remains unknown.

## Data Availability

Data is contained within the article or Supplementary Material.

## Supplementary Materials

The following are available online at https://www.mdpi.com/article/10.3390/cancers13184614/s1; Figure S1 Full Western blot images; Figure S2: Full confocal images; Figure S3: Additional experimental data; Table S1: Raw experimental data and full statistical analysis.
