# Supplementary material for: CBP/p300 Bromodomain Inhibitor–I–CBP112 Declines Transcription of the Key ABC Transporters and Sensitizes Cancer Cells to Chemotherapy Drugs
Source: Cancers (Basel). 2021 Sep 14;13(18):4614. doi: 10.3390/cancers13184614 (PMC8467251; doi:10.3390/cancers13184614)
Supplement: Supplementary file 1 [file cancers-13-04614-s001.zip › Figure S2.pptx]

## Slide 1
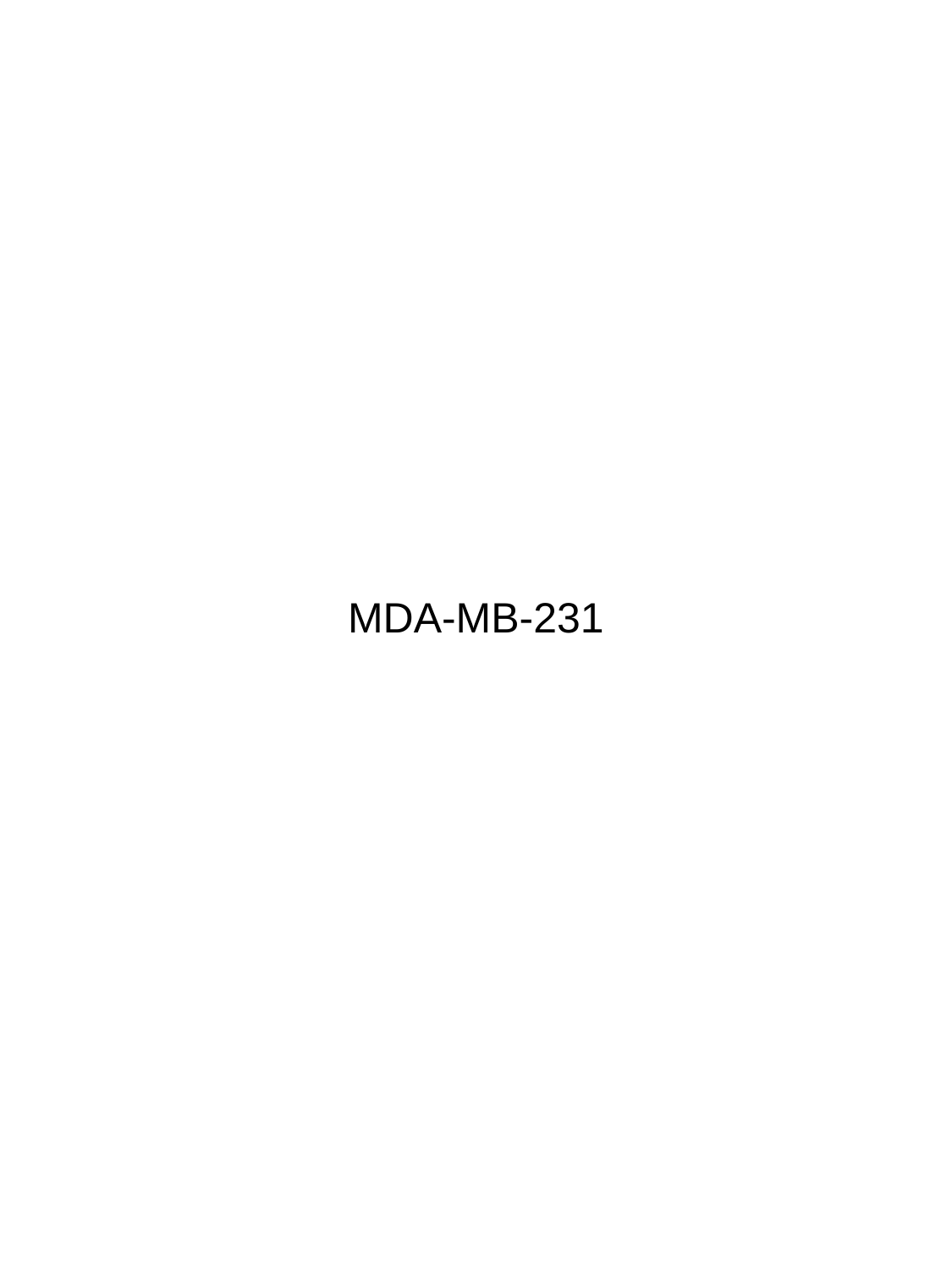

# MDA-MB-231

## Slide 2
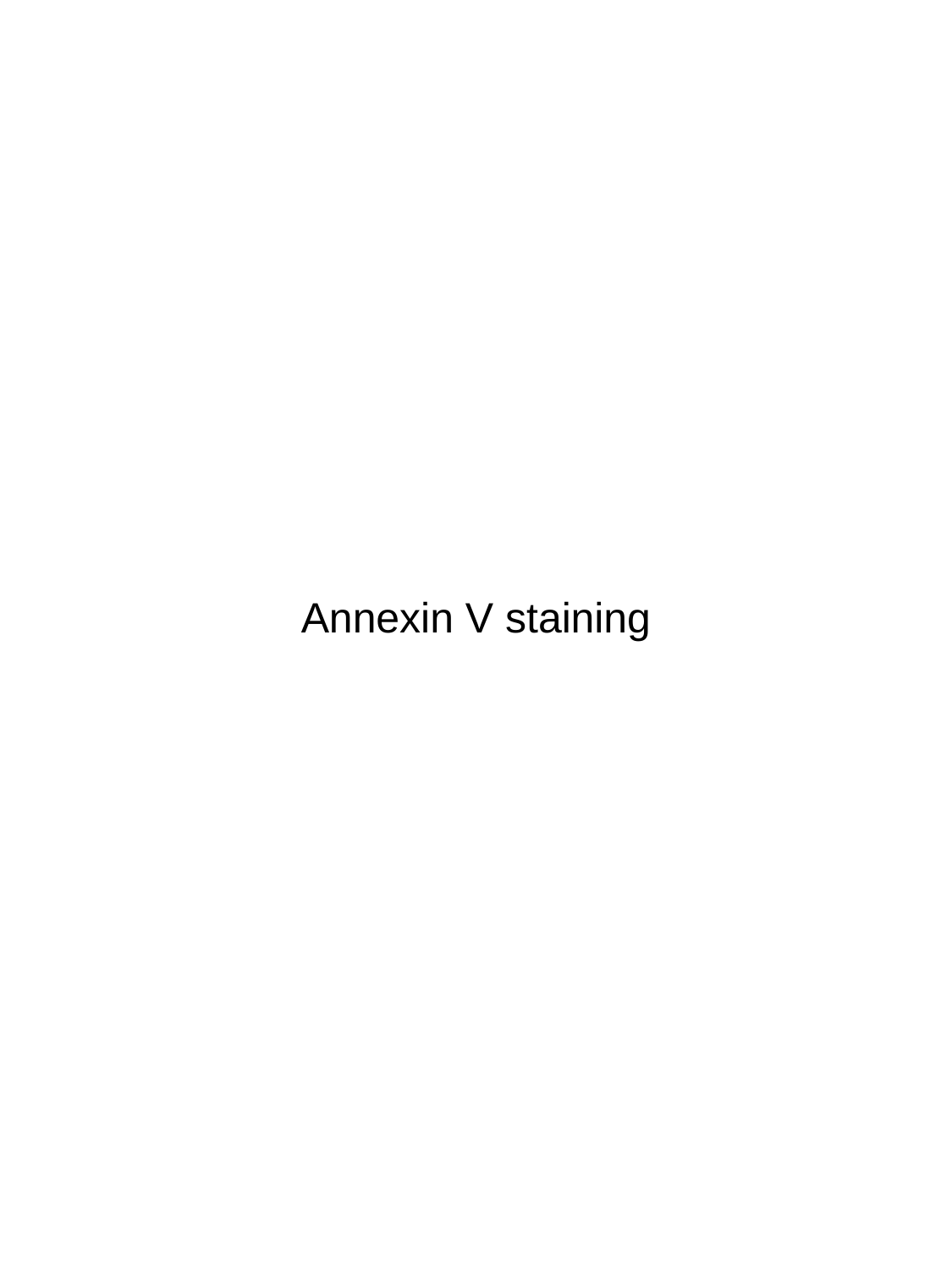

# Annexin V staining

## Slide 3
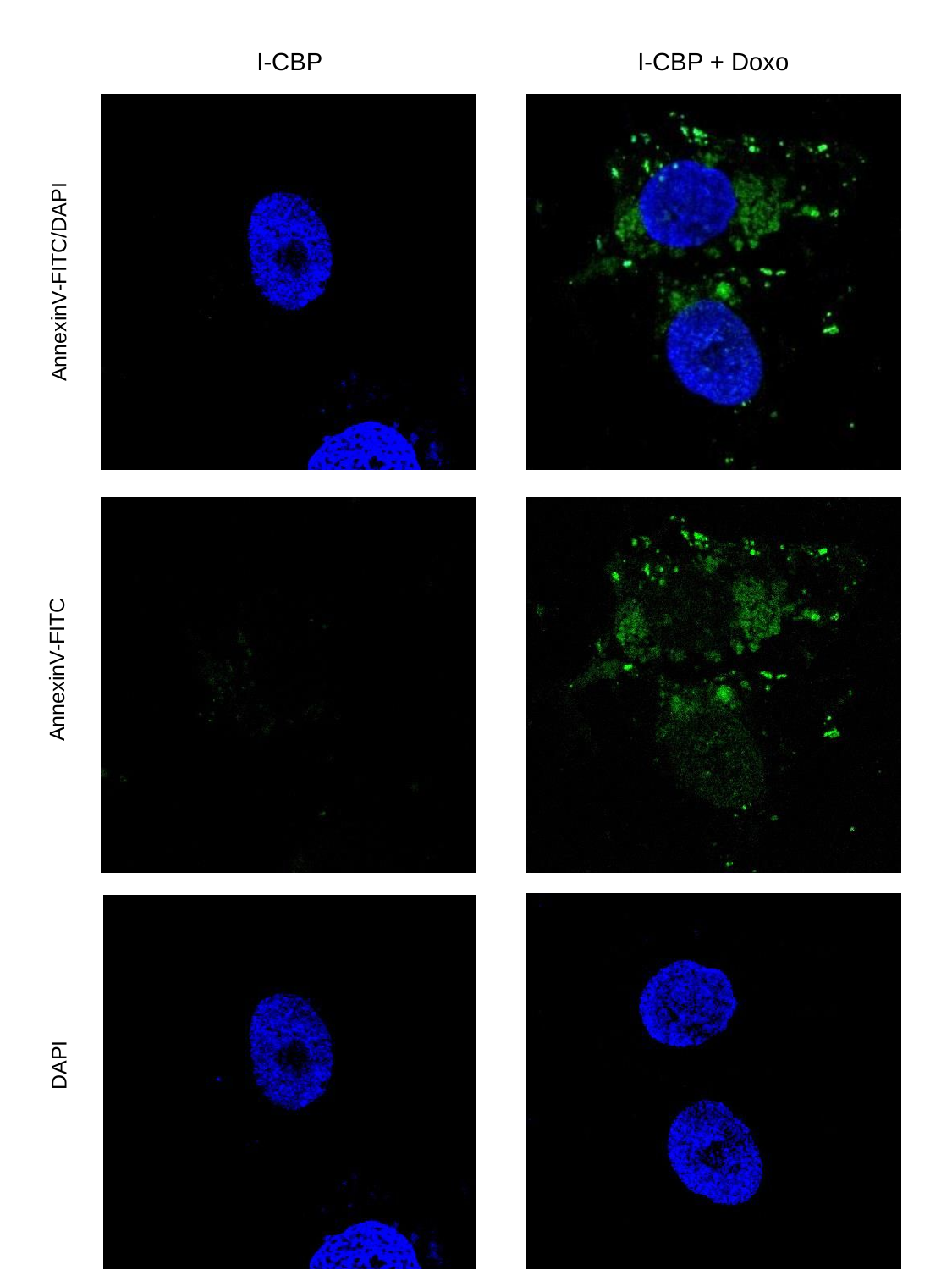

I-CBP + Doxo
I-CBP
AnnexinV-FITC/DAPI
AnnexinV-FITC
DAPI

## Slide 4
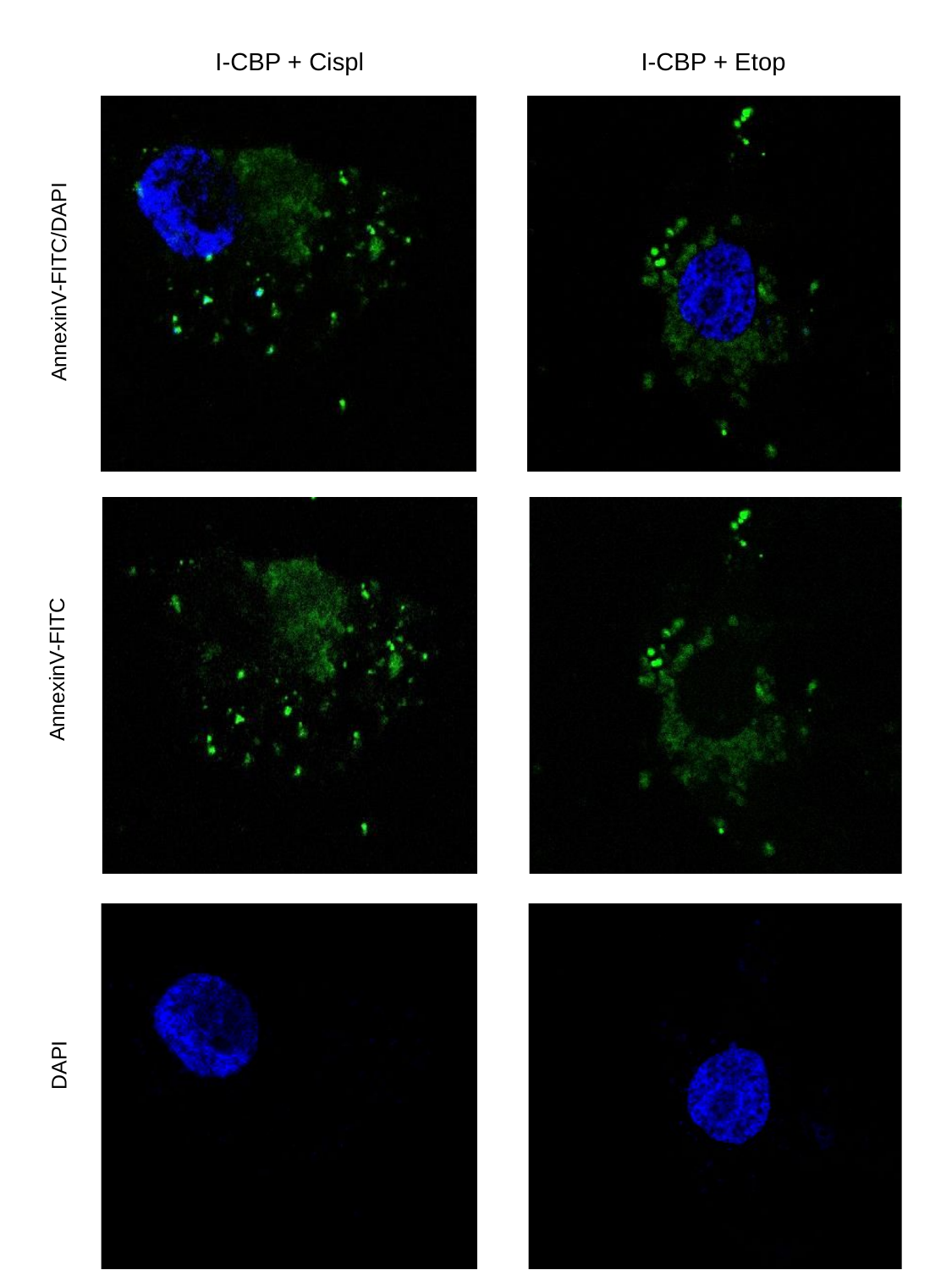

I-CBP + Cispl
I-CBP + Etop
AnnexinV-FITC/DAPI
AnnexinV-FITC
DAPI

## Slide 5
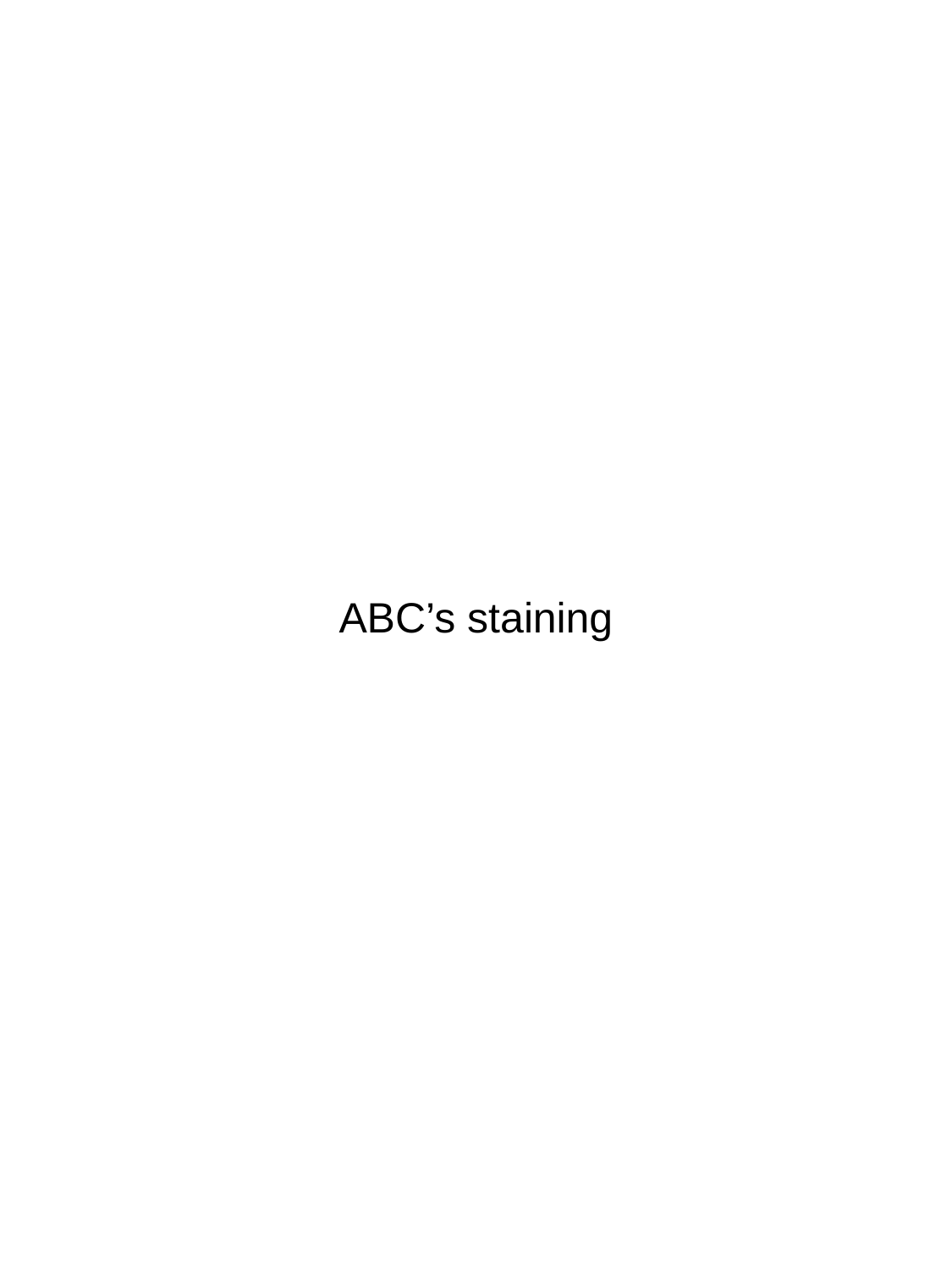

# ABC’s staining

## Slide 6
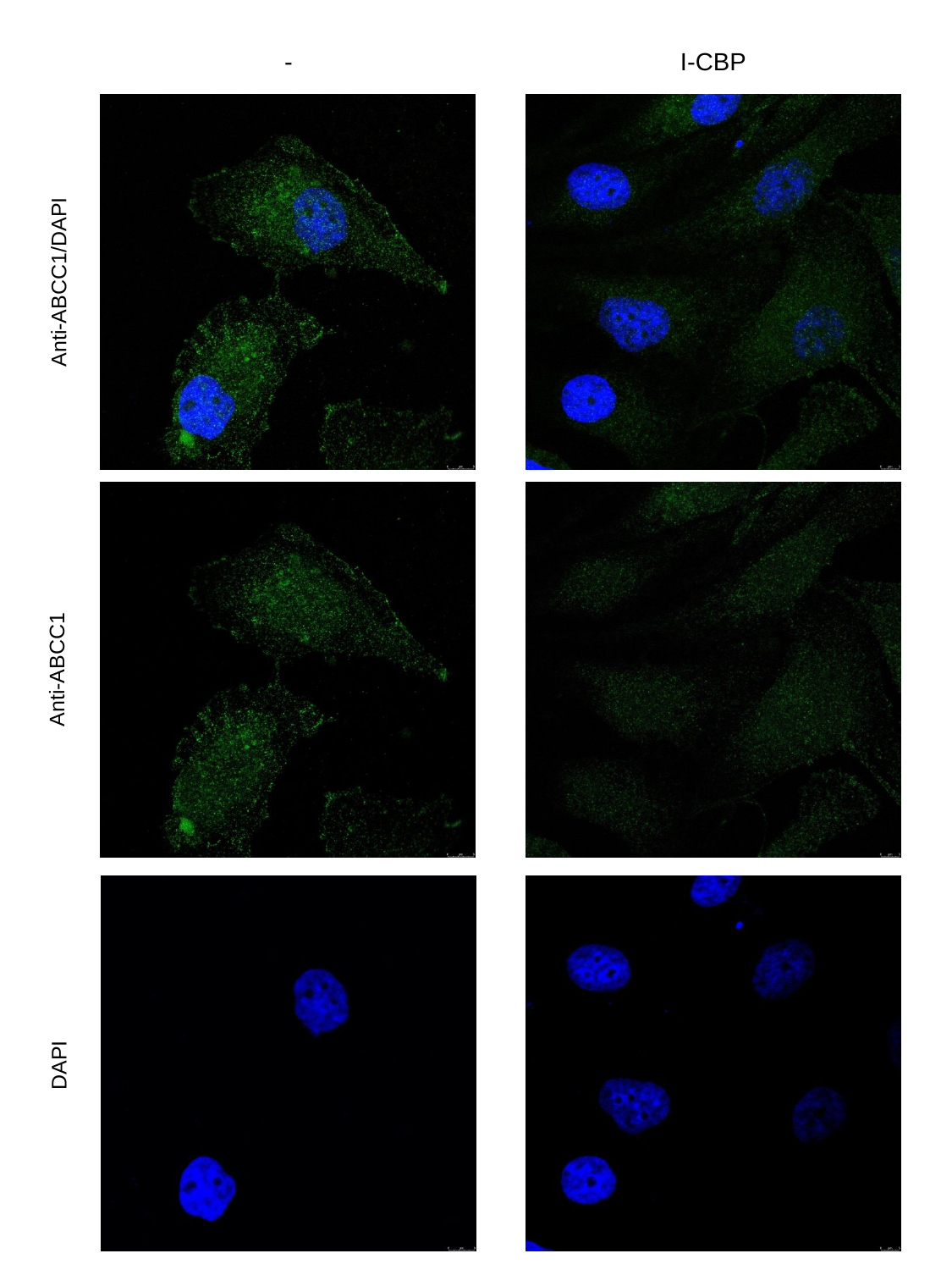

-
I-CBP
Anti-ABCC1/DAPI
Anti-ABCC1
DAPI

## Slide 7
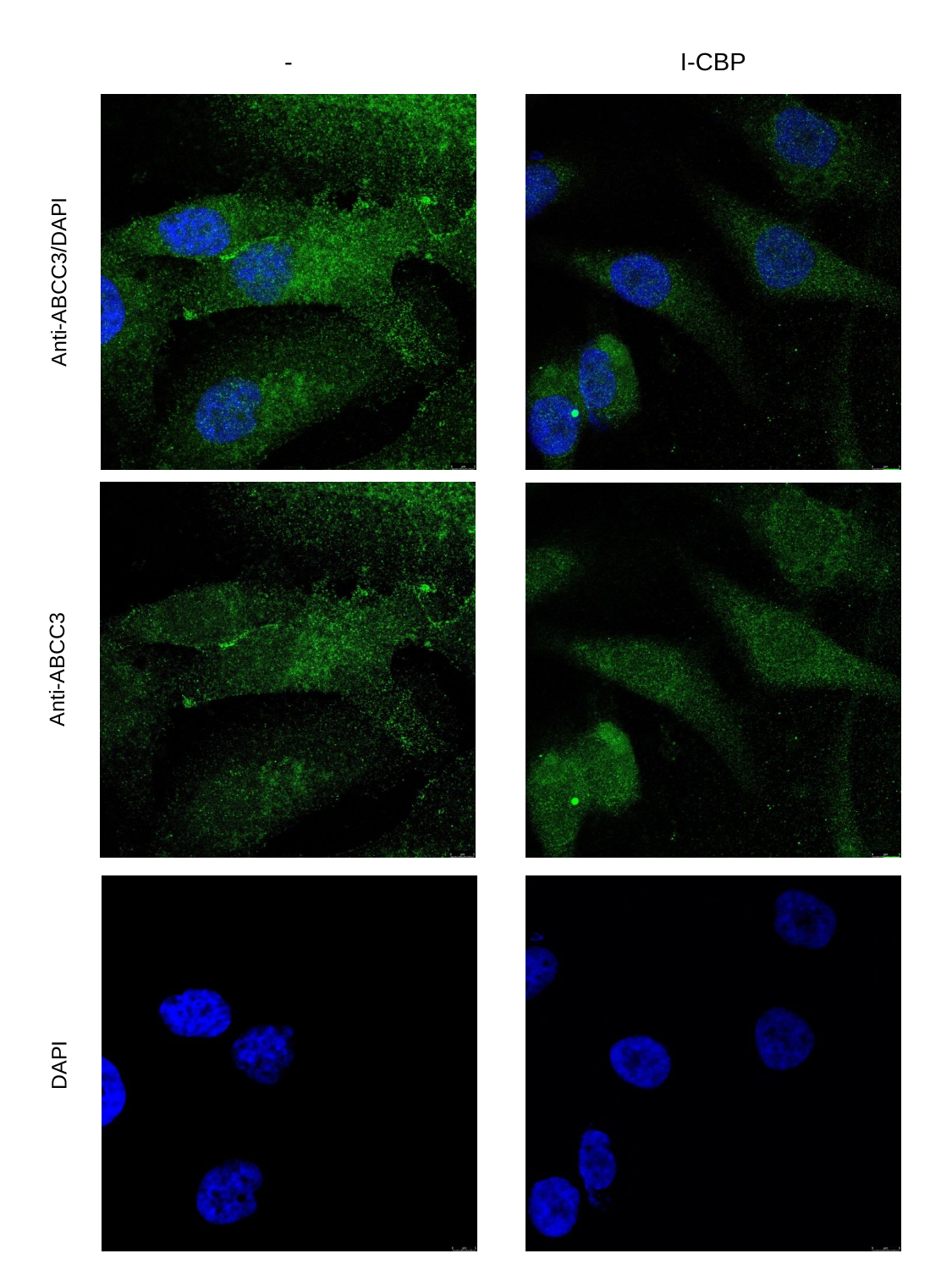

-
I-CBP
Anti-ABCC3/DAPI
Anti-ABCC3
DAPI

## Slide 8
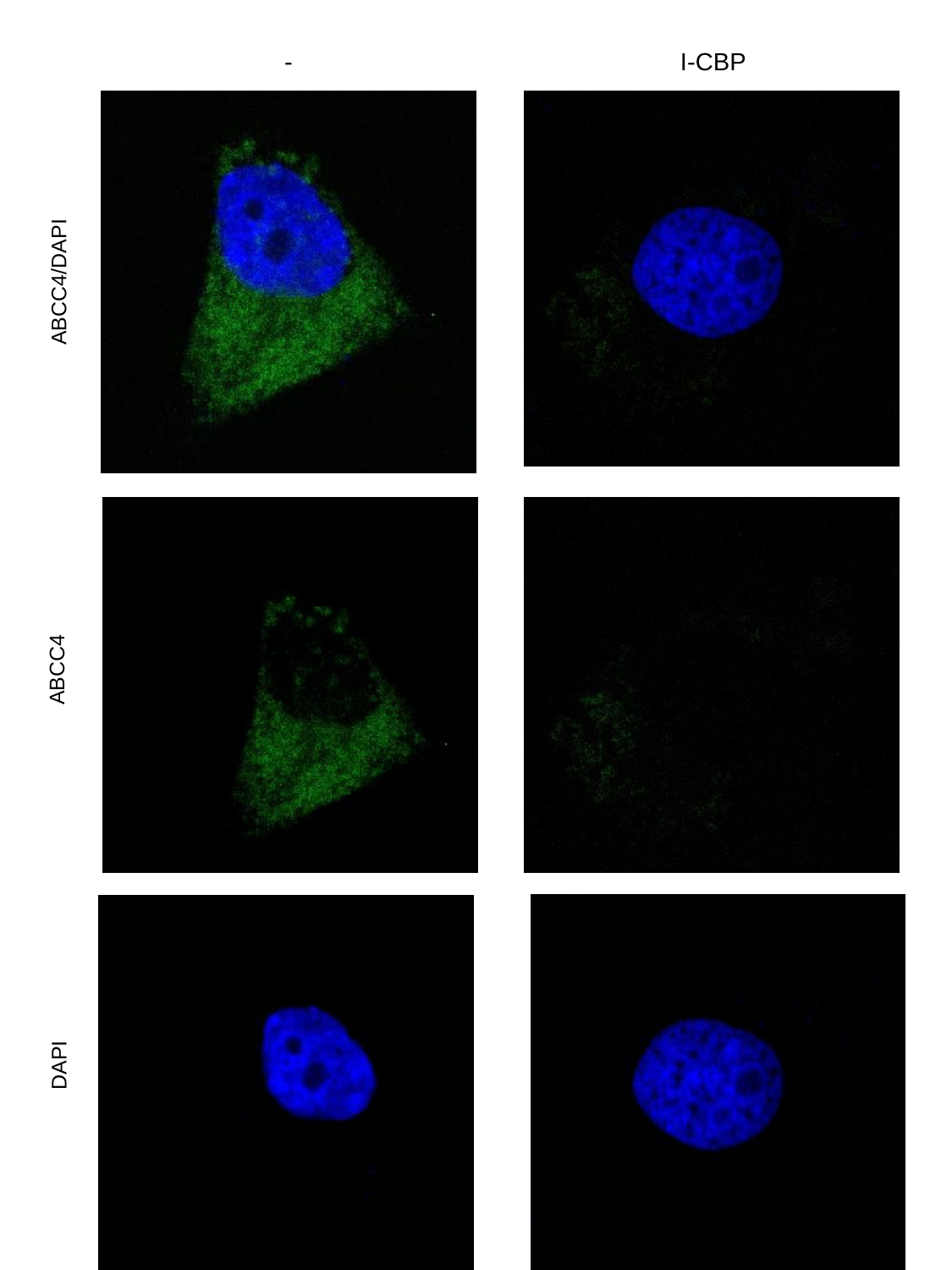

-
I-CBP
ABCC4/DAPI
ABCC4
DAPI

## Slide 9
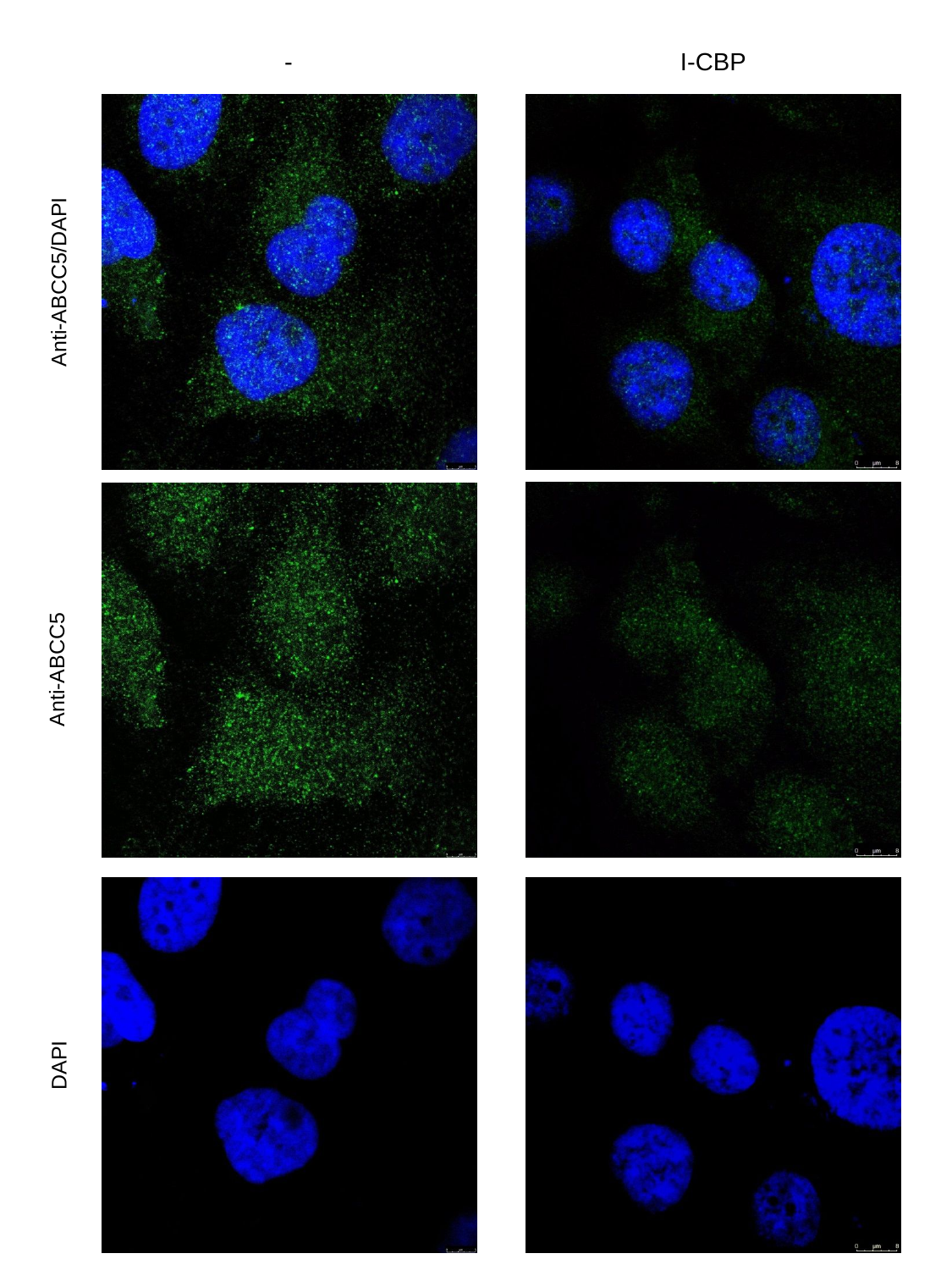

-
I-CBP
Anti-ABCC5/DAPI
Anti-ABCC5
DAPI

## Slide 10
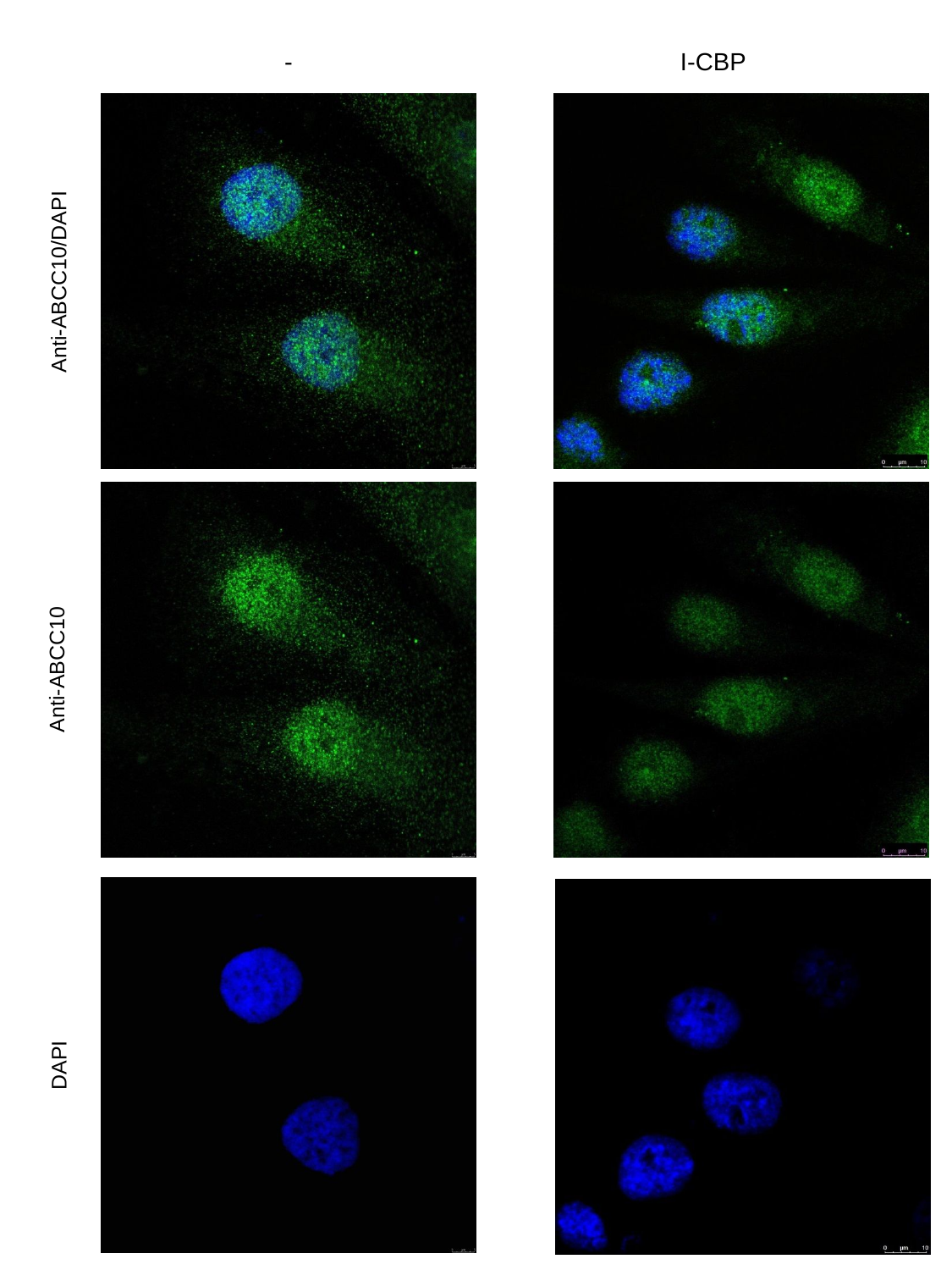

-
I-CBP
Anti-ABCC10/DAPI
Anti-ABCC10
DAPI

## Slide 11
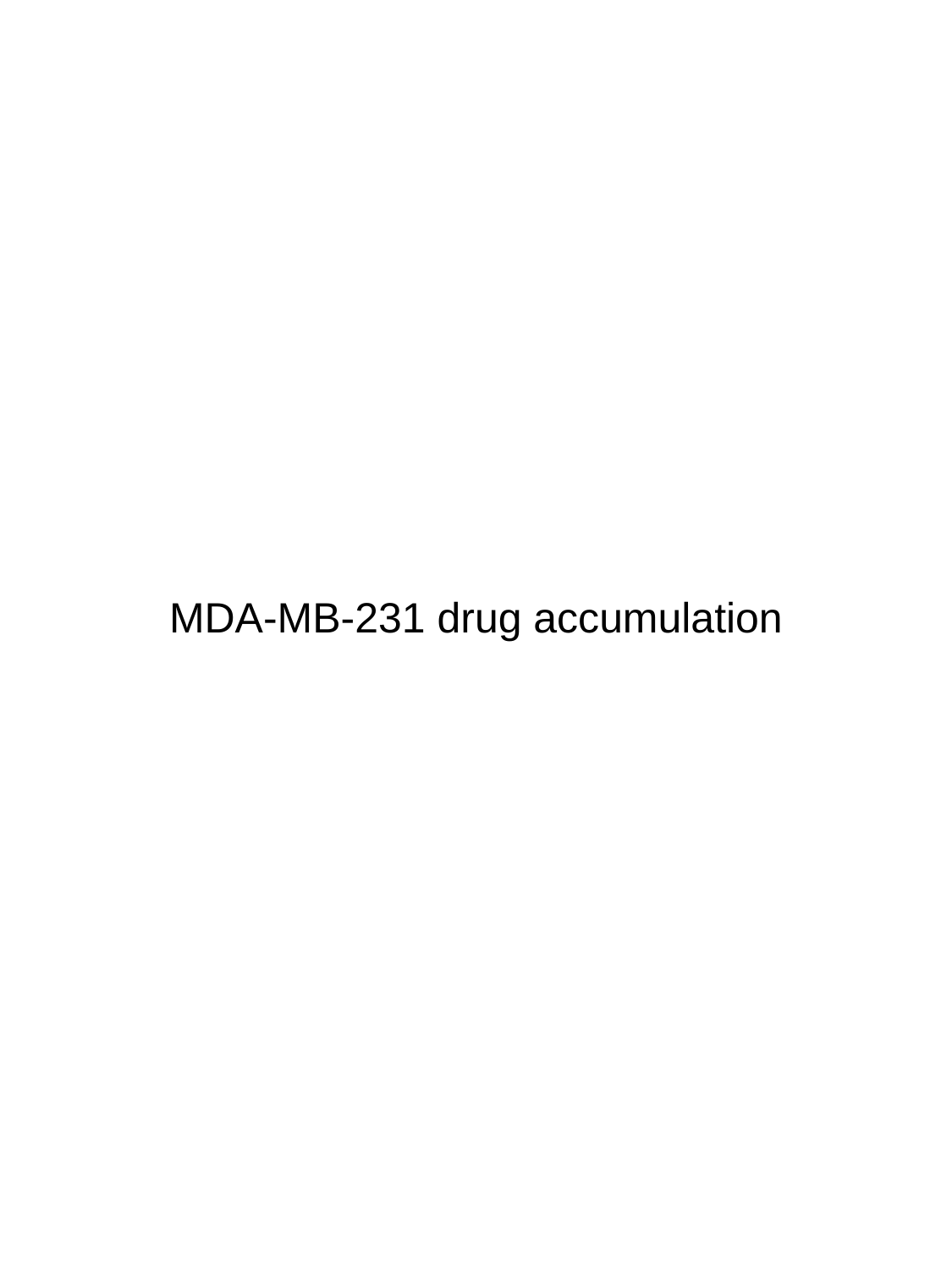

# MDA-MB-231 drug accumulation

## Slide 12
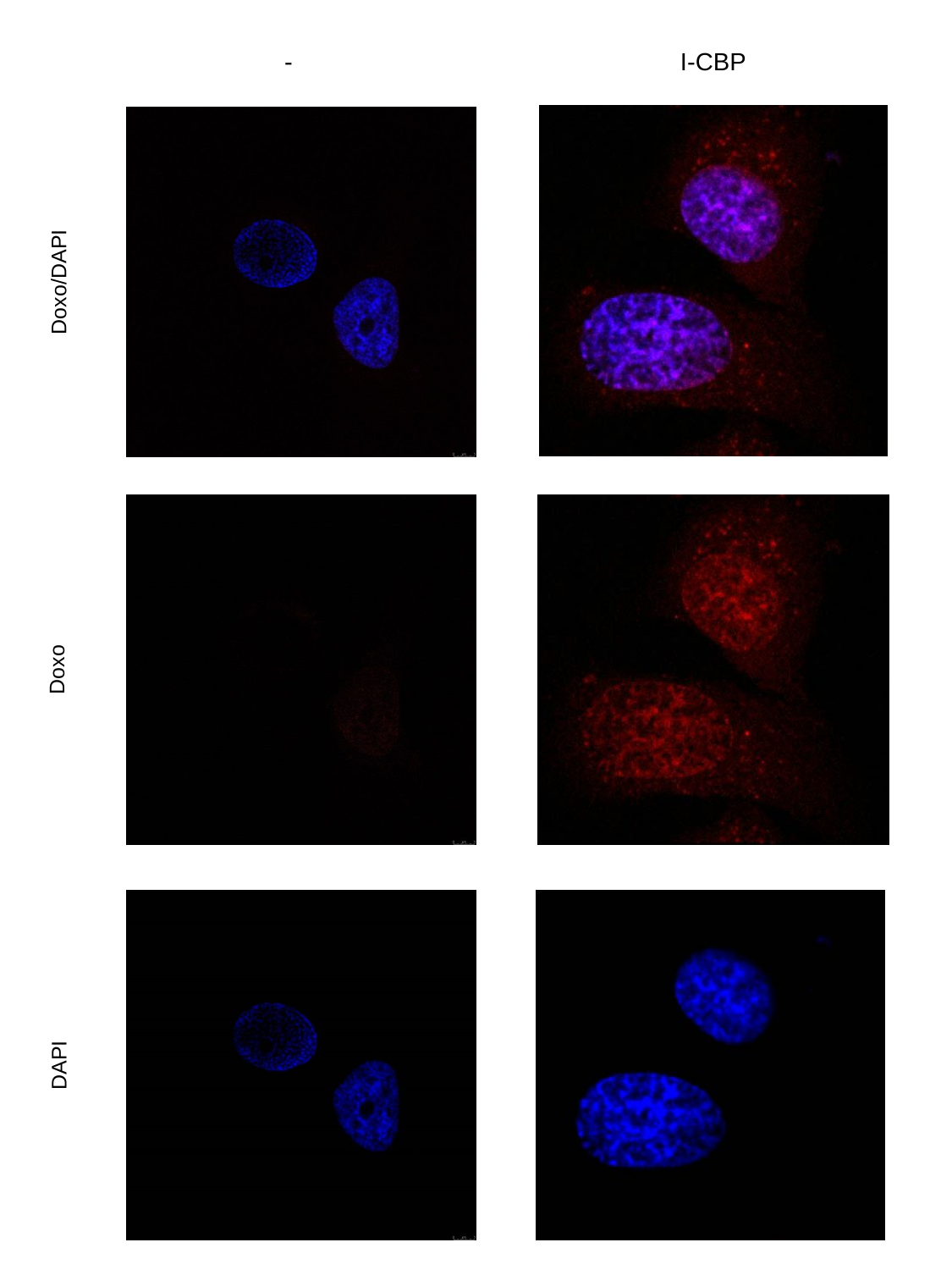

-
I-CBP
Doxo/DAPI
Doxo
DAPI

## Slide 13
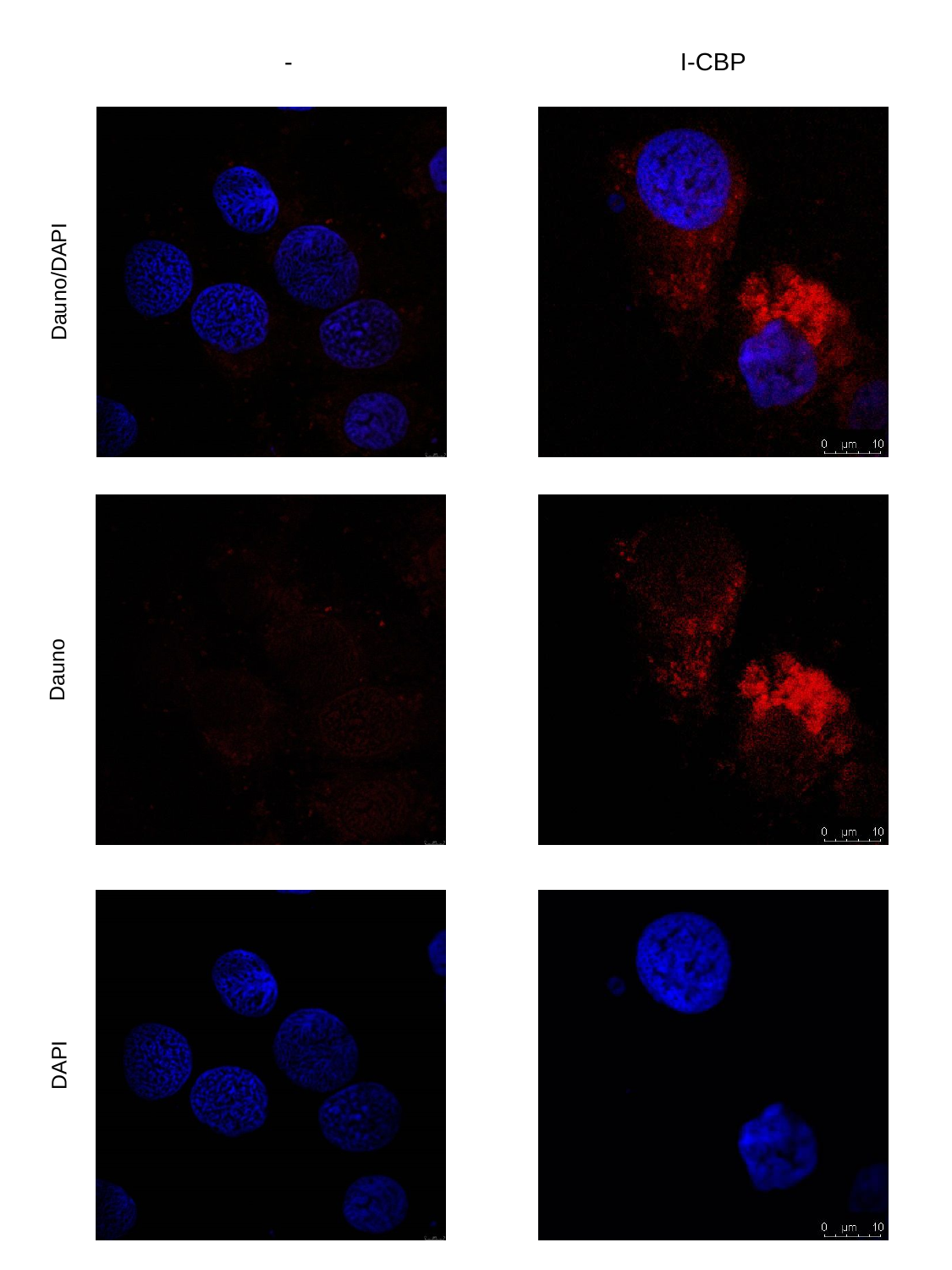

-
I-CBP
Dauno/DAPI
Dauno
DAPI

## Slide 14
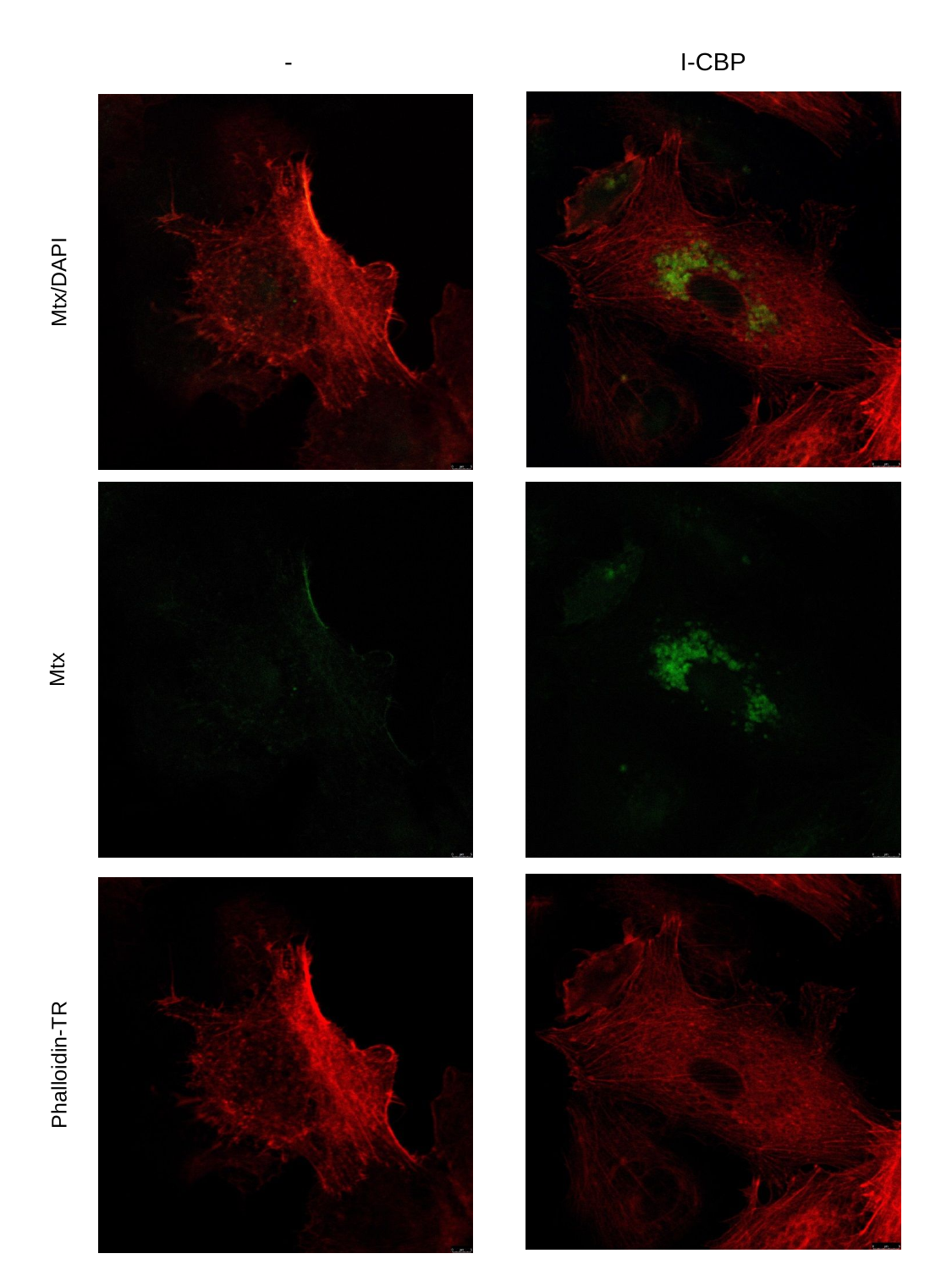

-
I-CBP
Mtx/DAPI
Mtx
Phalloidin-TR

## Slide 15
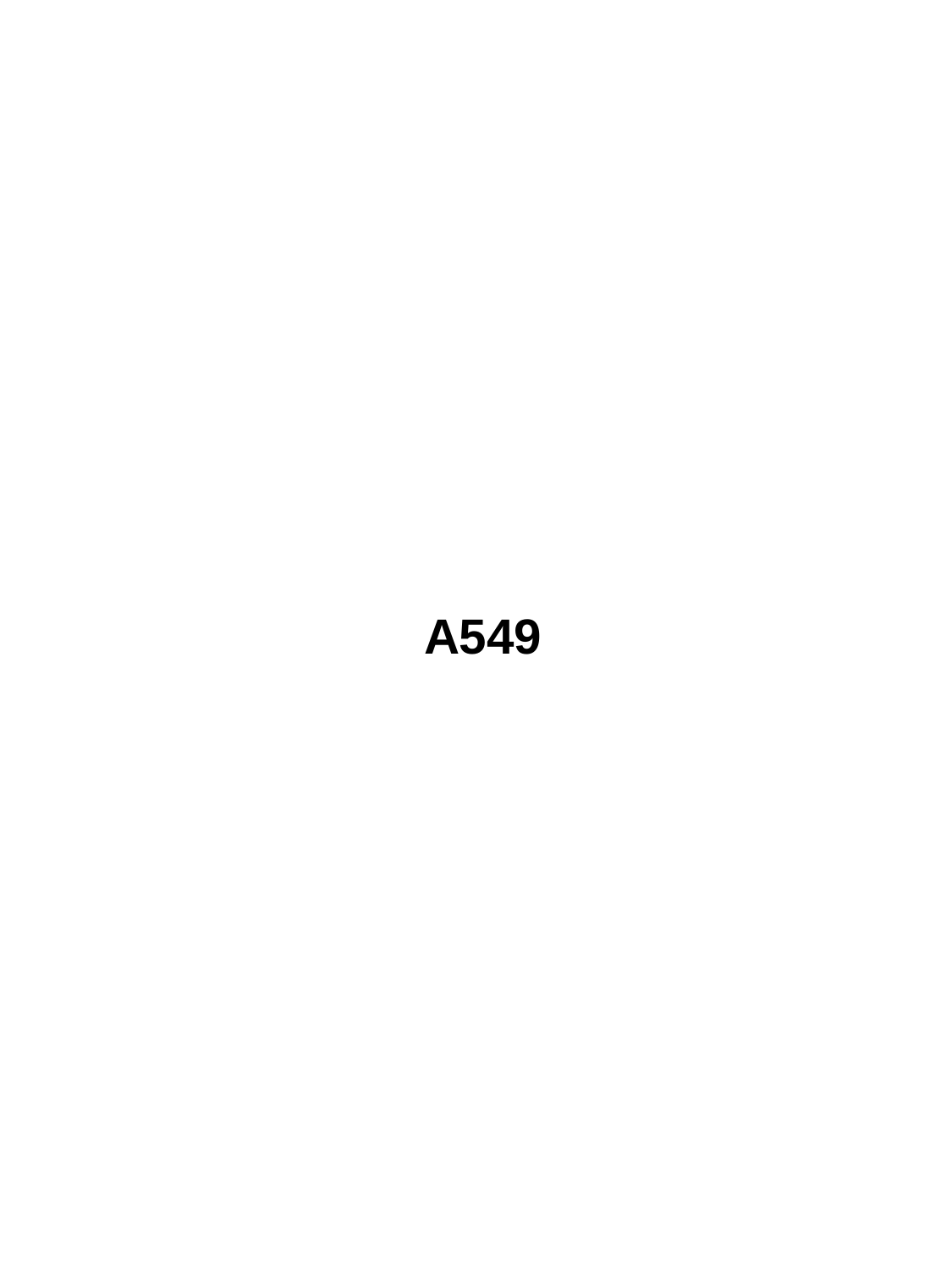

A549

## Slide 16
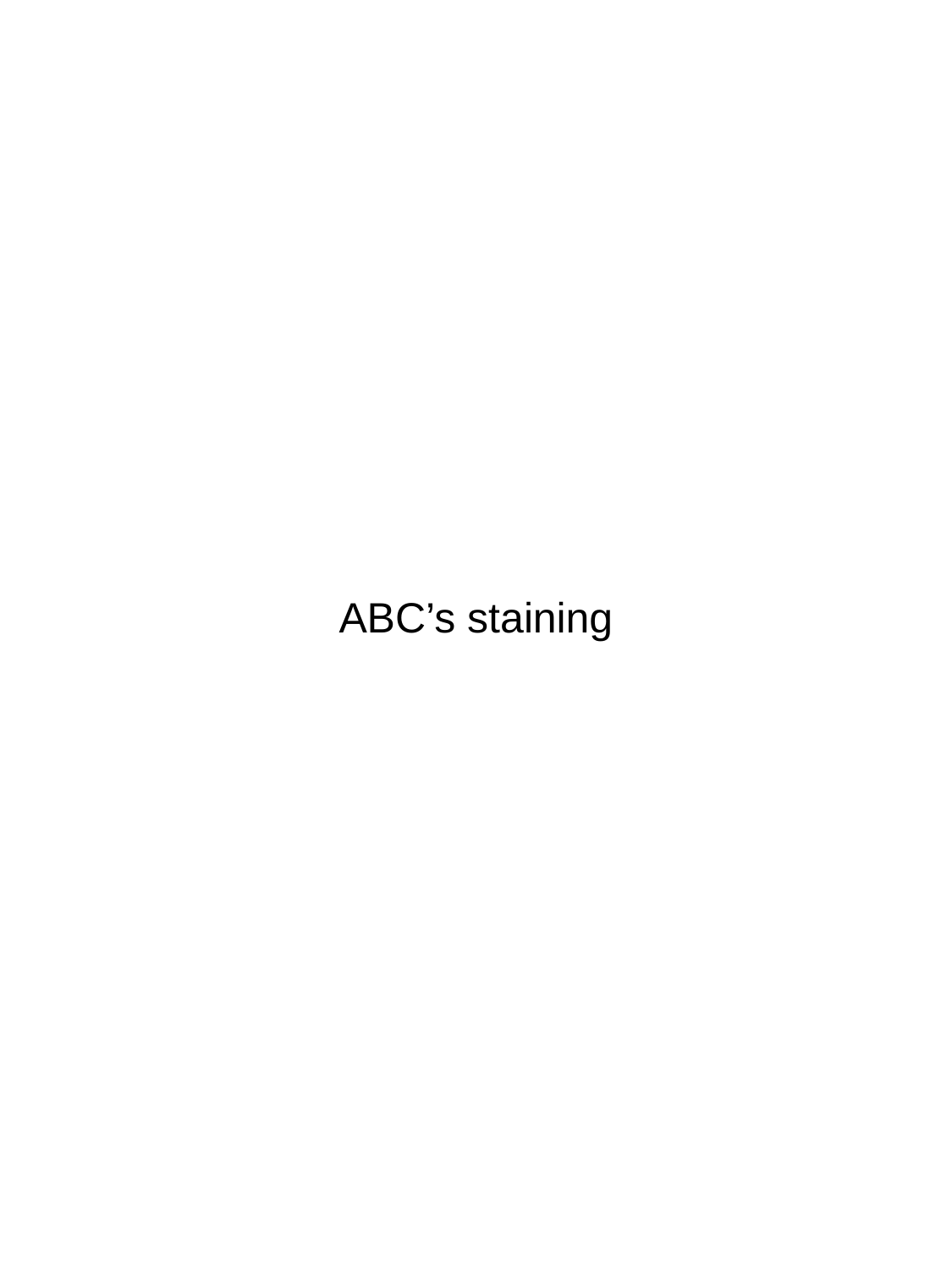

# ABC’s staining

## Slide 17
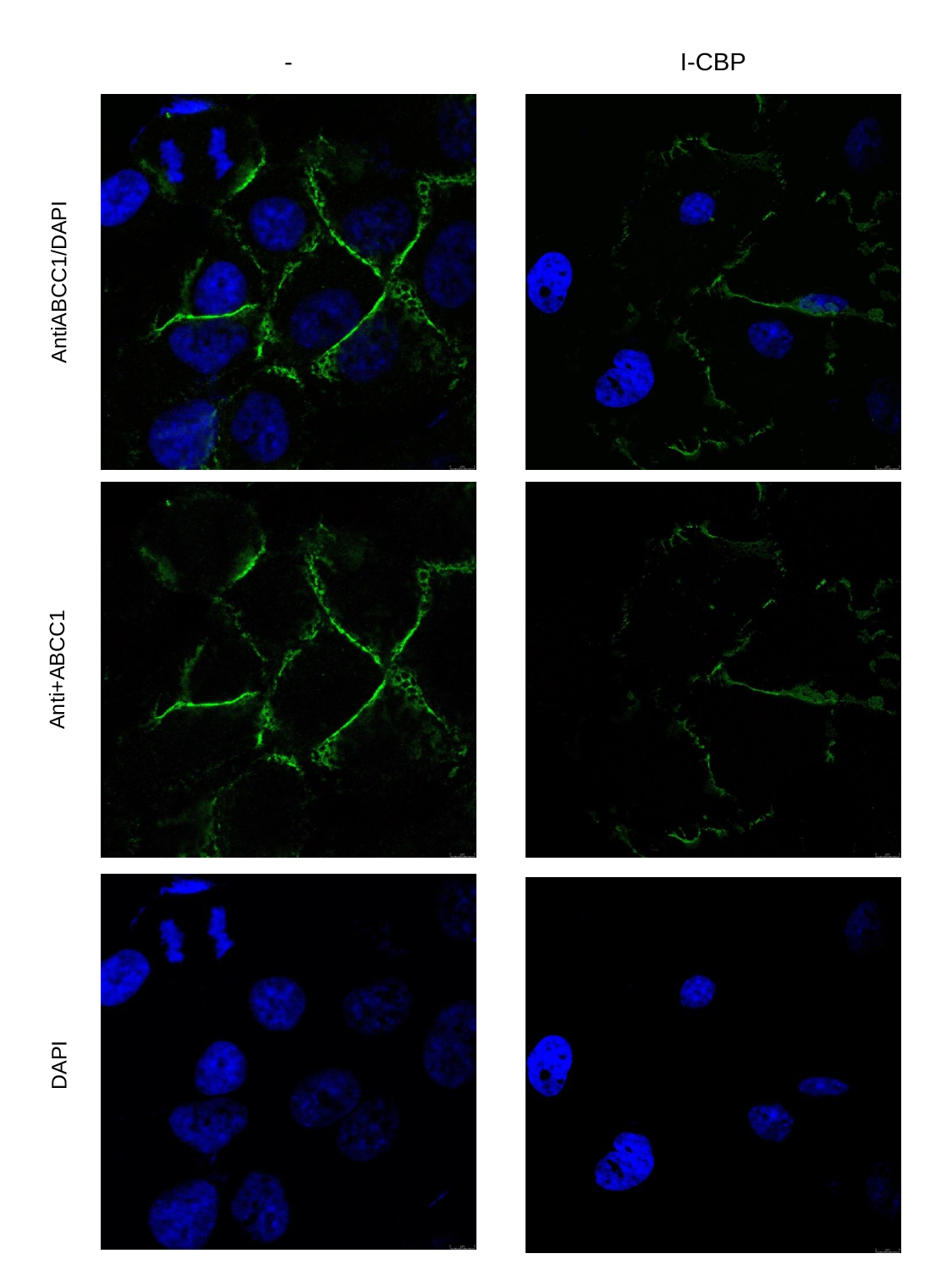

-
I-CBP
AntiABCC1/DAPI
Anti+ABCC1
DAPI

## Slide 18
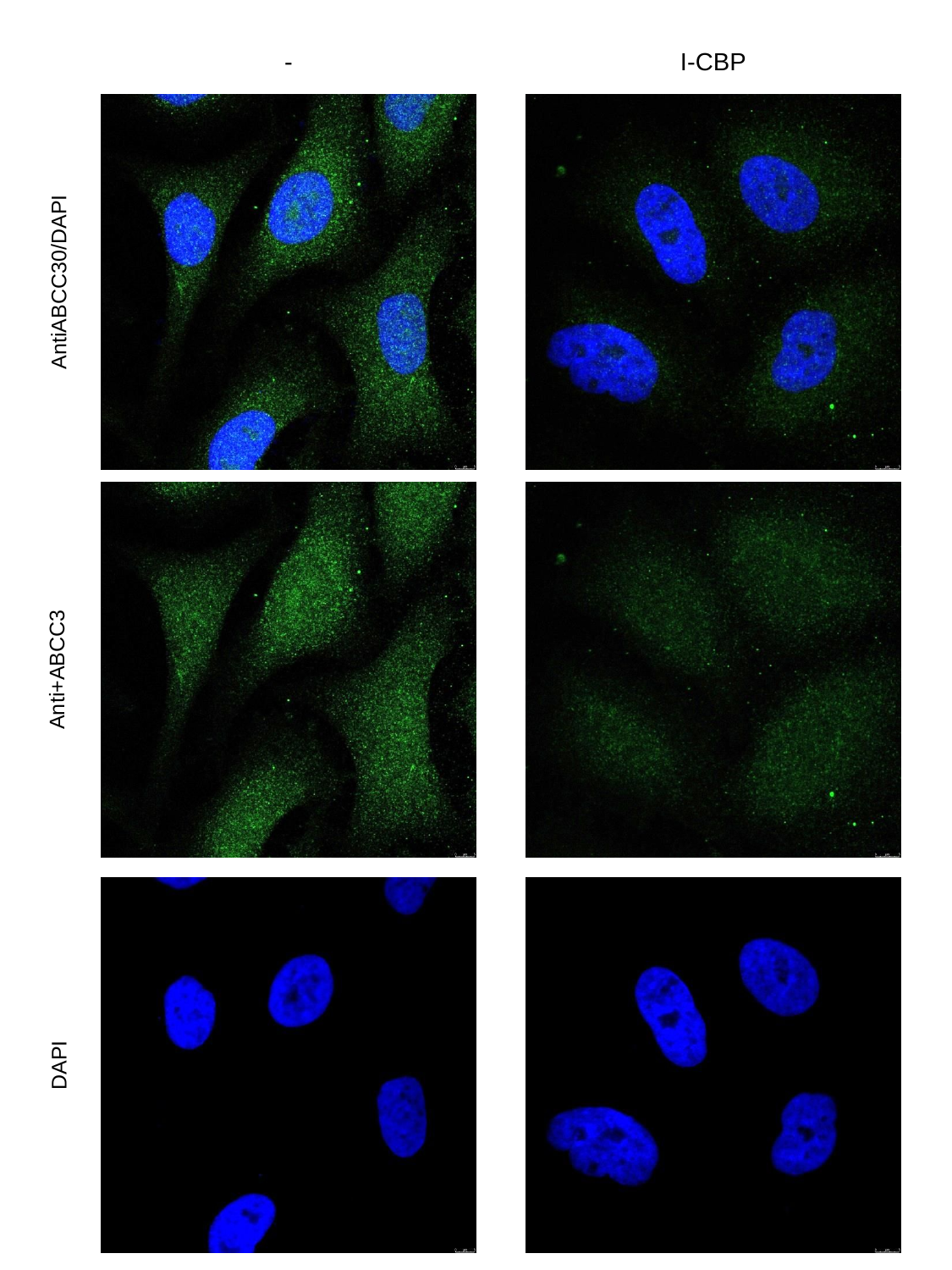

-
I-CBP
AntiABCC30/DAPI
Anti+ABCC3
DAPI

## Slide 19
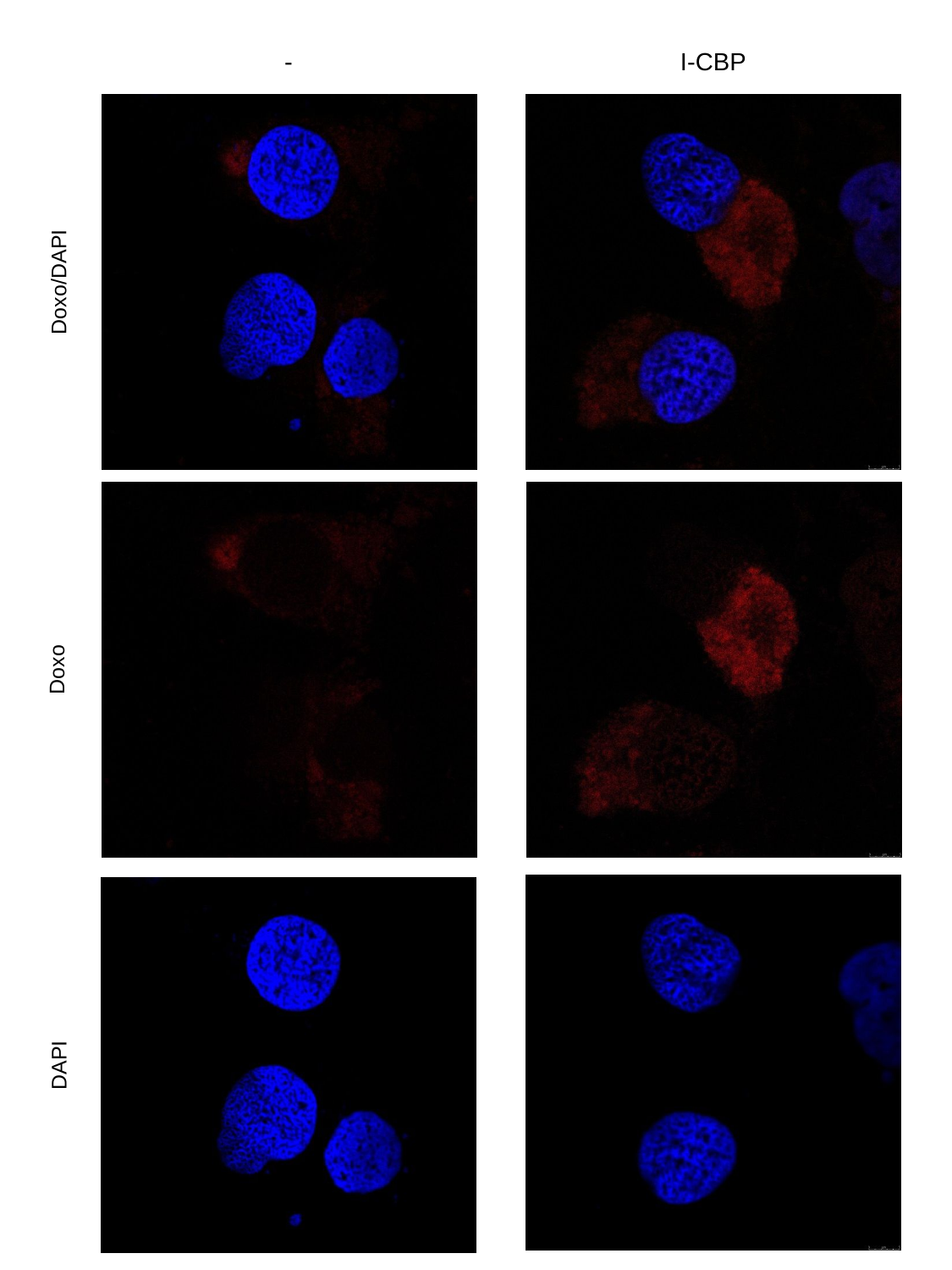

-
I-CBP
Doxo/DAPI
Doxo
DAPI

## Slide 20
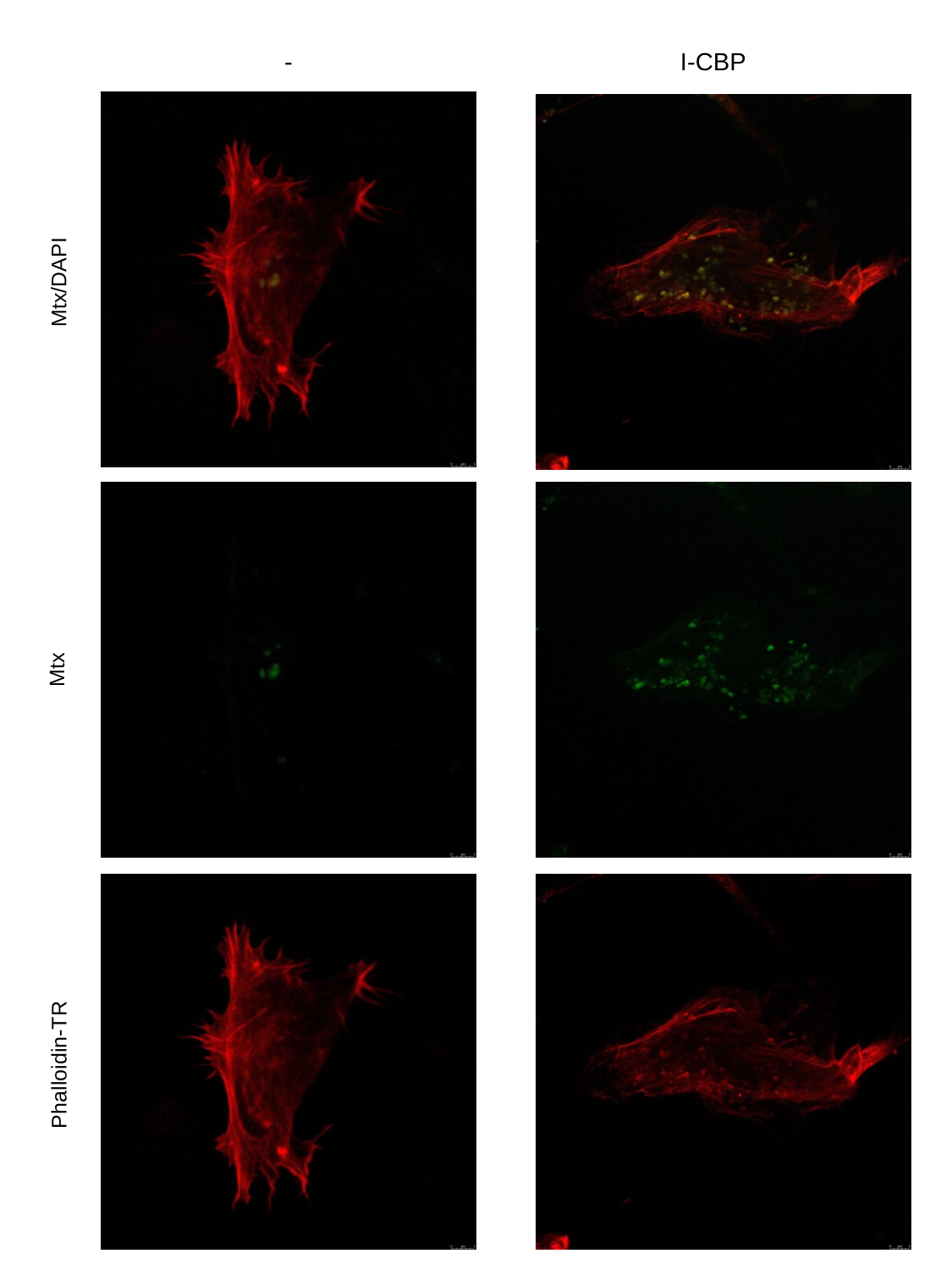

-
I-CBP
Mtx/DAPI
Mtx
Phalloidin-TR

## Slide 21
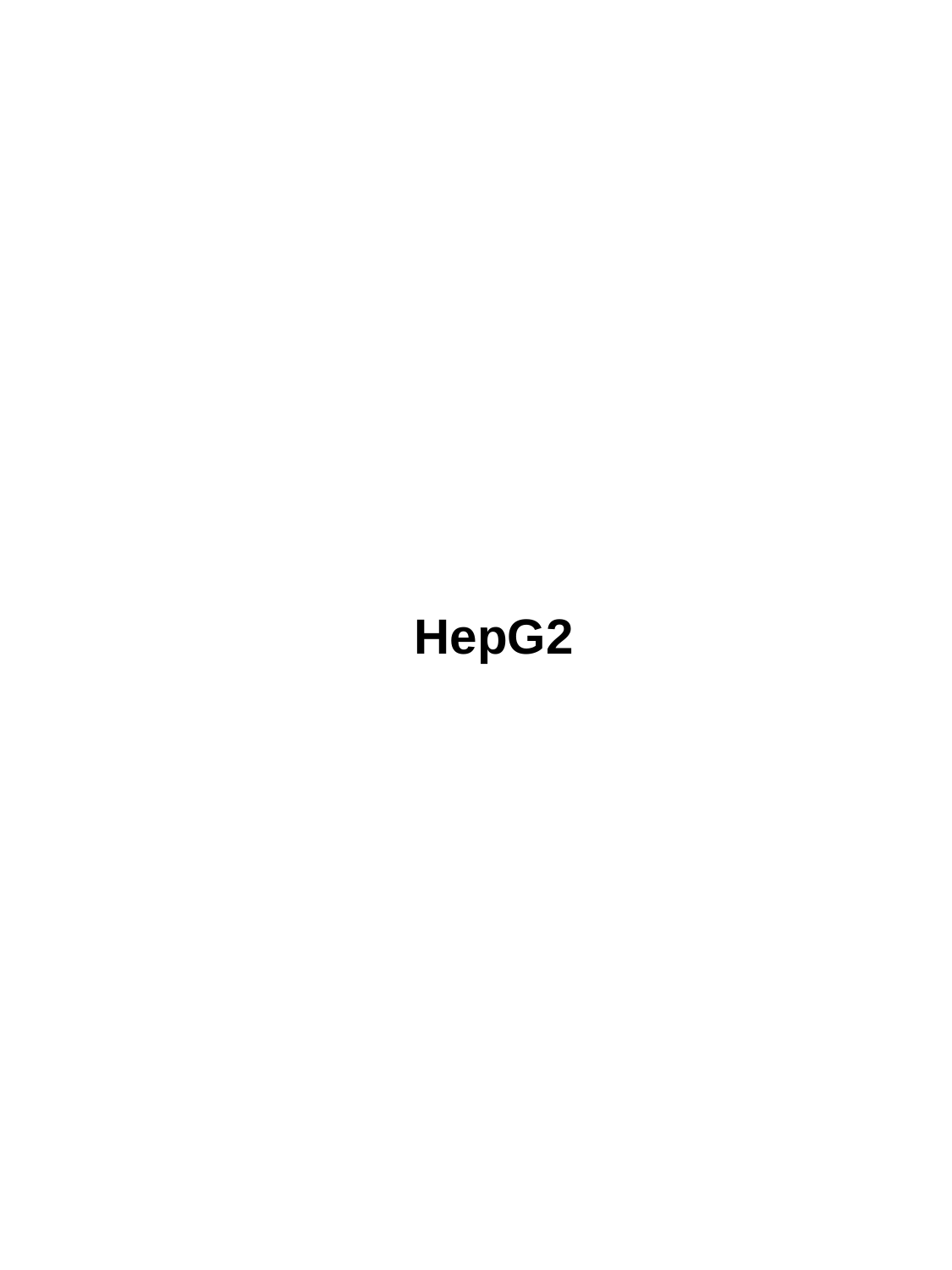

HepG2

## Slide 22
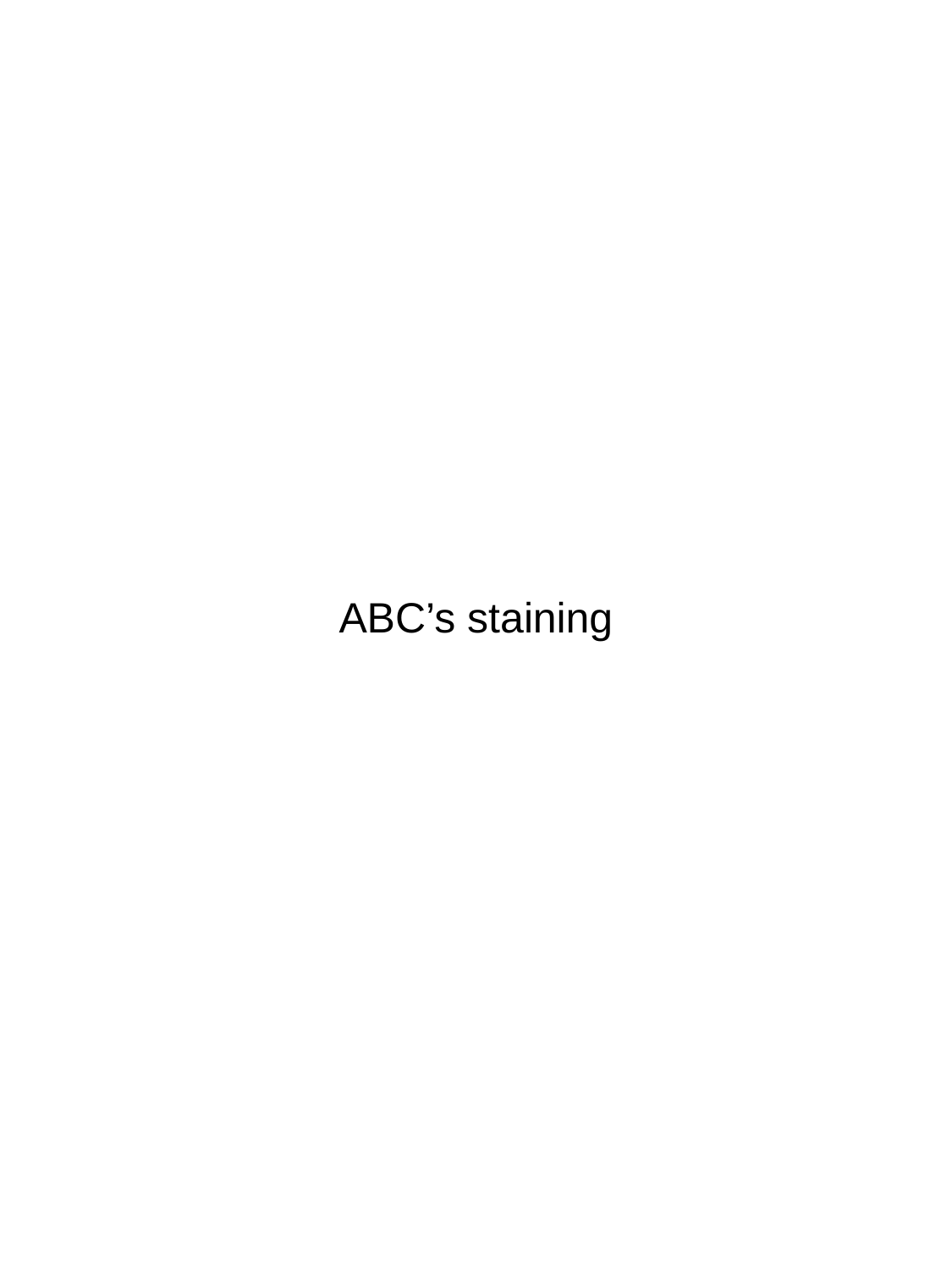

# ABC’s staining

## Slide 23
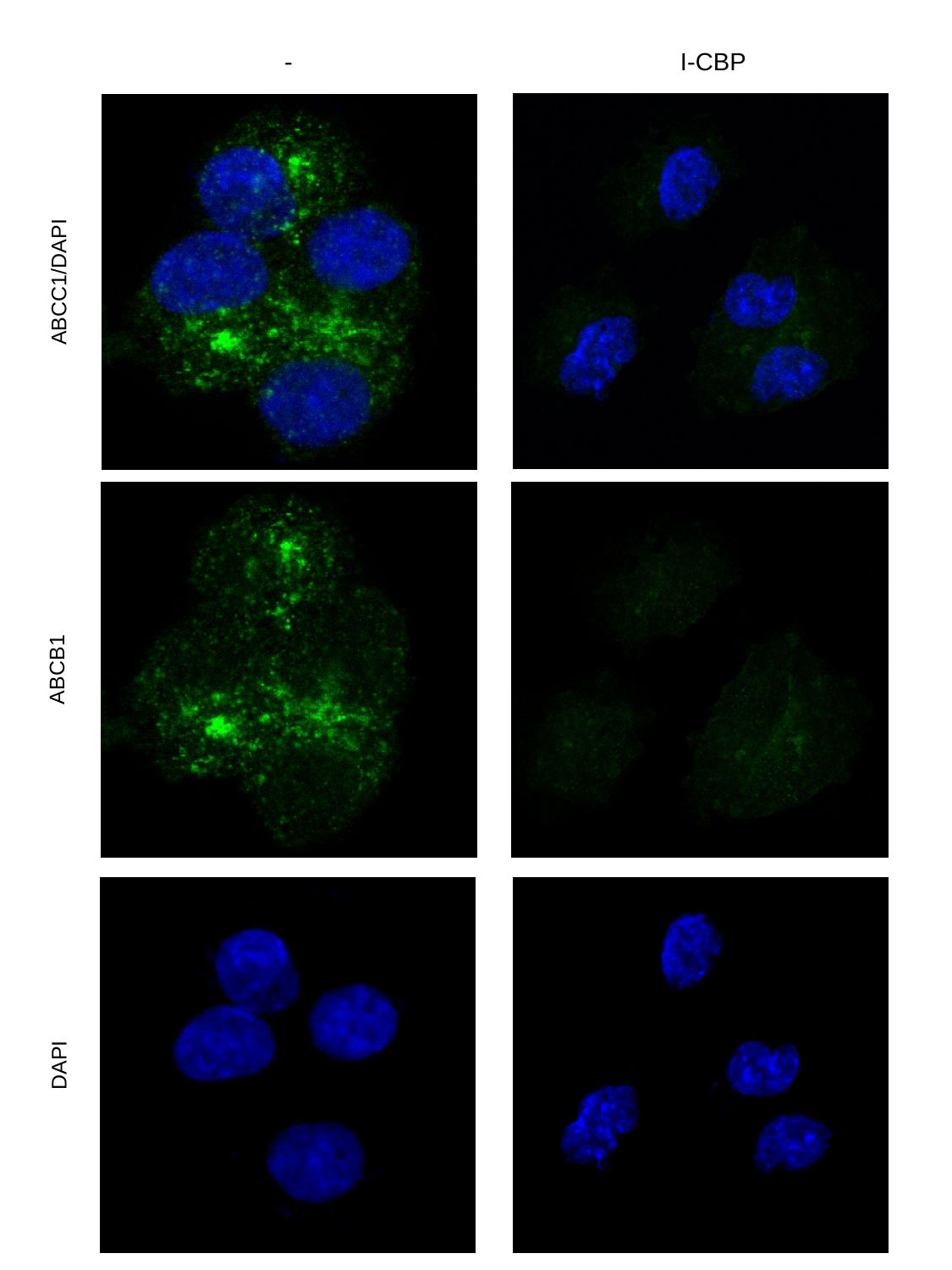

-
I-CBP
ABCC1/DAPI
ABCB1
DAPI

## Slide 24
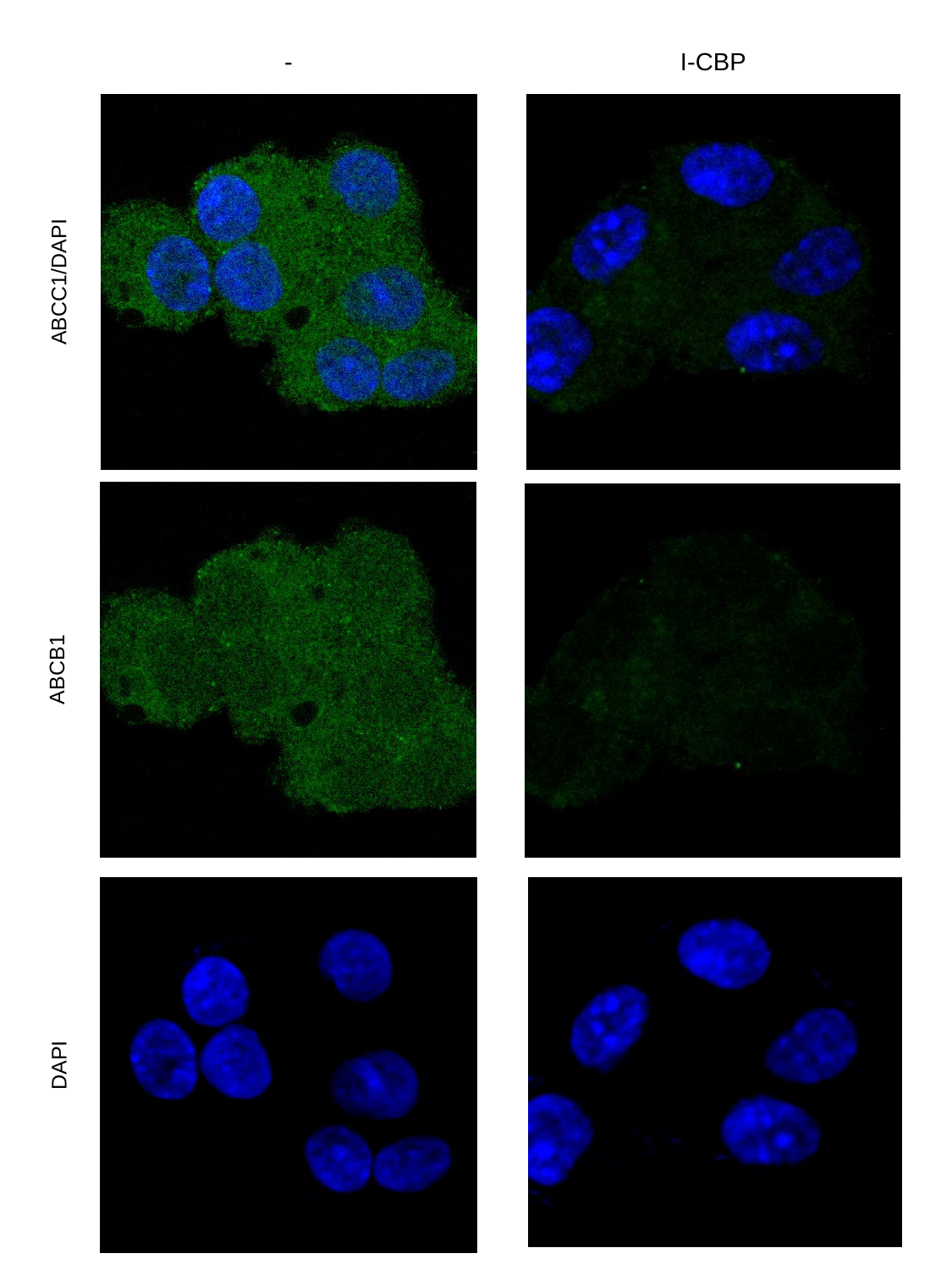

-
I-CBP
ABCC1/DAPI
ABCB1
DAPI

## Slide 25
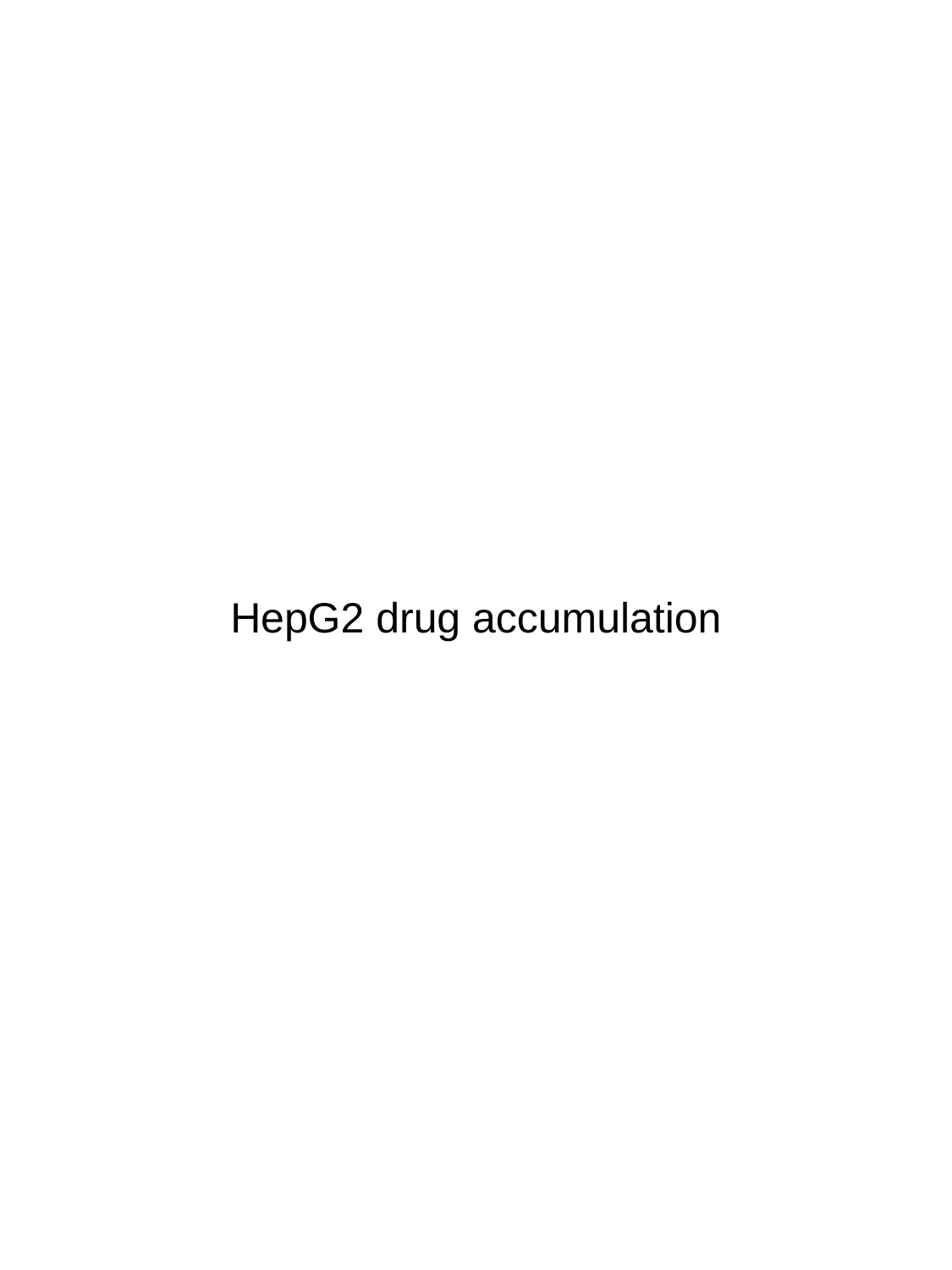

# HepG2 drug accumulation

## Slide 26
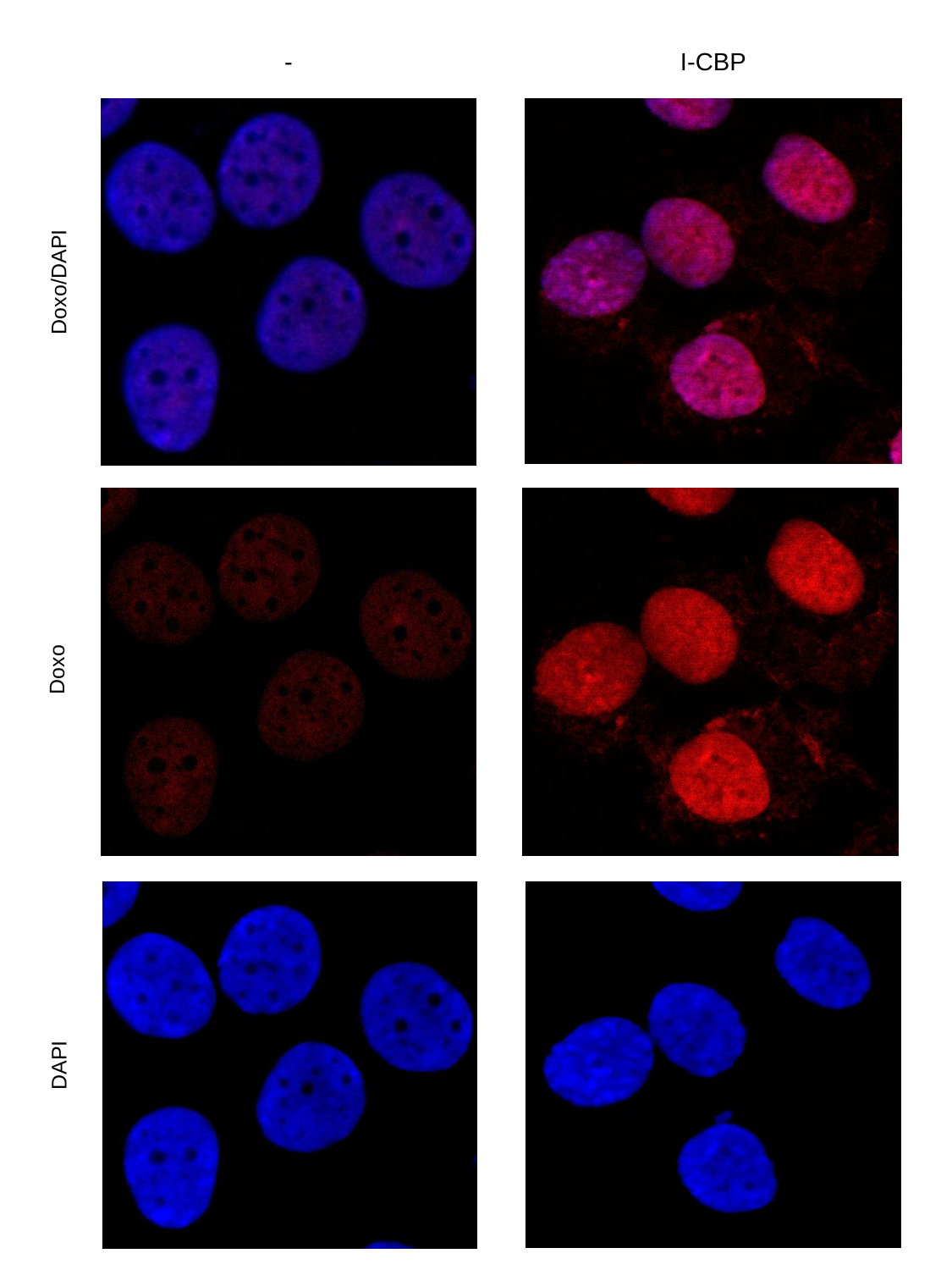

-
I-CBP
Doxo/DAPI
Doxo
DAPI

## Slide 27
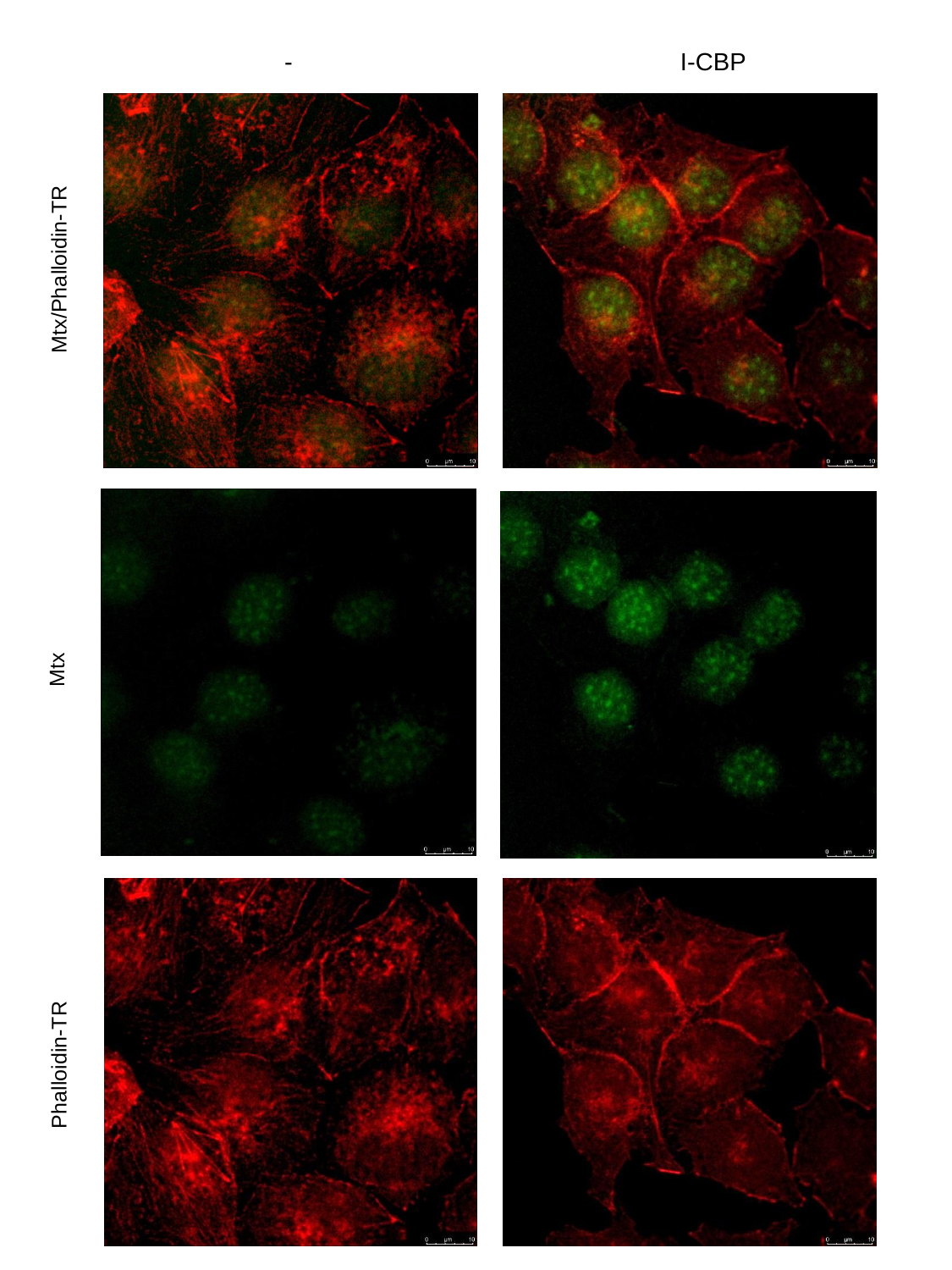

-
I-CBP
Mtx/Phalloidin-TR
Mtx
Phalloidin-TR
