# Supplementary material for: CBP/p300 Bromodomain Inhibitor–I–CBP112 Declines Transcription of the Key ABC Transporters and Sensitizes Cancer Cells to Chemotherapy Drugs
Source: Cancers (Basel). 2021 Sep 14;13(18):4614. doi: 10.3390/cancers13184614 (PMC8467251; doi:10.3390/cancers13184614)
Supplement: Supplementary file 1 [file cancers-13-04614-s001.zip › Figure S3.pptx]

## Slide 1
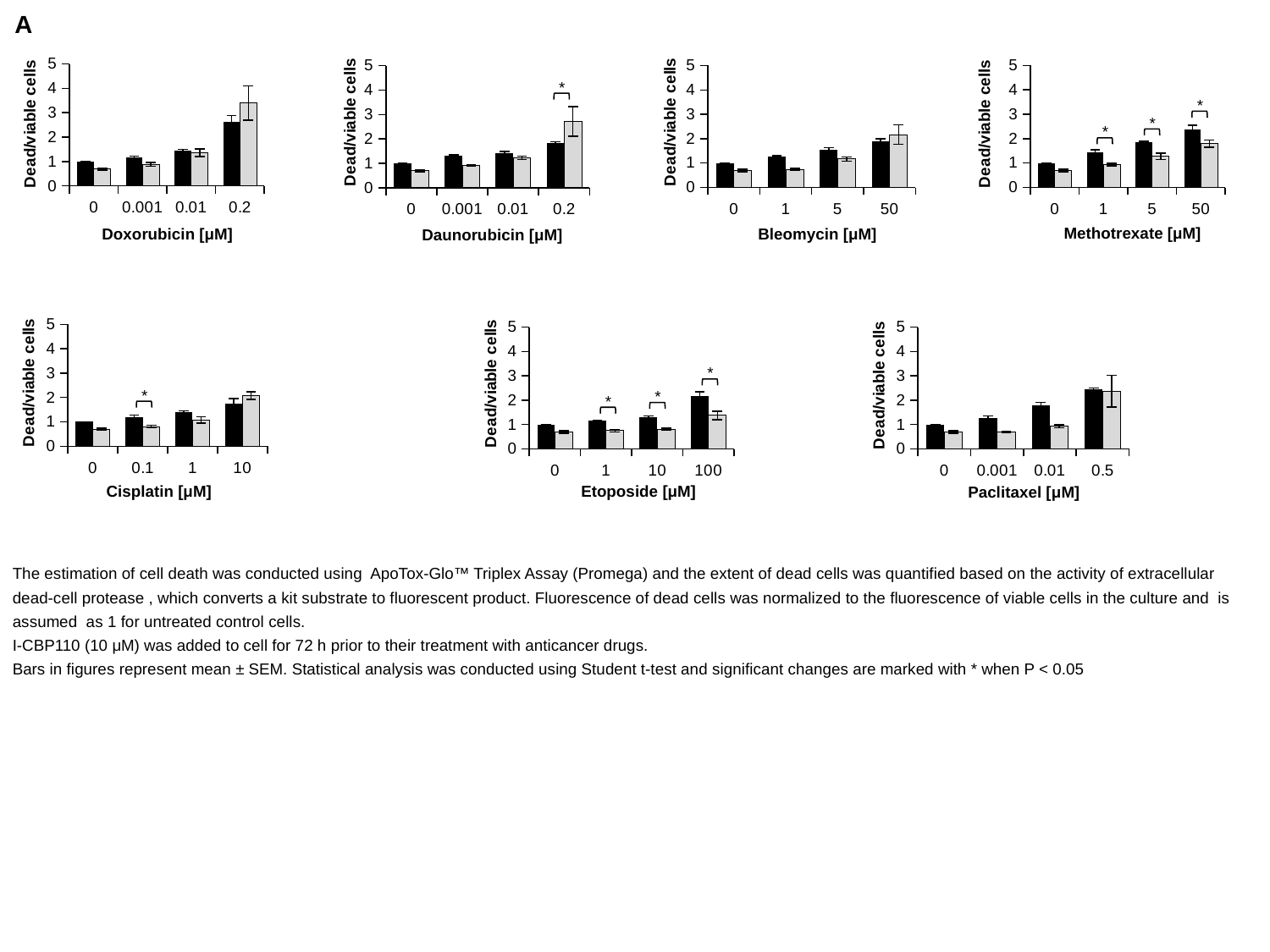

A
### Chart
| Category | | |
|---|---|---|
| 0 | 1.0003531073446328 | 0.6938401707948112 |
| 1E-3 | 1.1809341966243516 | 0.877548000207347 |
| 0.01 | 1.4311352180546049 | 1.3621448527155497 |
| 0.2 | 2.624464291103152 | 3.392815902364652 |Doxorubicin [μM]
### Chart
| Category | | |
|---|---|---|
| 0 | 1.0003531073446328 | 0.6938401707948124 |
| 1 | 1.4470638373909062 | 0.9318551520601629 |
| 5 | 1.843075424480023 | 1.2699027684721238 |
| 50 | 2.3613672369468786 | 1.7936254136333396 |Methotrexate [μM]
### Chart
| Category | | |
|---|---|---|
| 0 | 1.0003531073446328 | 0.6938401707948112 |
| 1 | 1.2585801405596664 | 0.7464585127911012 |
| 5 | 1.5615864595889195 | 1.1666591487019184 |
| 50 | 1.8780217542406996 | 2.1696454310857987 |Bleomycin [μM]
### Chart
| Category | | |
|---|---|---|
| 0 | 1.0003531073446328 | 0.6938401707948112 |
| 1E-3 | 1.2926444451554713 | 0.9023591879689412 |
| 0.01 | 1.4232703015803163 | 1.2263237087849452 |
| 0.2 | 1.8179726571045127 | 2.7191892684832033 |Daunorubicin [μM]
*
*
*
*
### Chart
| Category | | |
|---|---|---|
| 0 | 1.0003531073446328 | 0.6938401707948112 |
| 0.1 | 1.1917593040039474 | 0.8087657580357556 |
| 1 | 1.4053663423016893 | 1.073635035877688 |
| 10 | 1.7610784302934985 | 2.076772064887336 |Cisplatin [μM]
### Chart
| Category | | |
|---|---|---|
| 0 | 1.0003531073446328 | 0.6938401707948112 |
| 1 | 1.1538476399013309 | 0.750746181197979 |
| 10 | 1.29215595509278 | 0.8166866529349509 |
| 100 | 2.182690719700092 | 1.3772676444240428 |
### Chart
| Category | | |
|---|---|---|
| 0 | 1.0003531073446328 | 0.6938401707948112 |
| 1E-3 | 1.2676154044856403 | 0.7118535607647697 |
| 0.01 | 1.7734690155329553 | 0.9257221565463787 |
| 0.5 | 2.438875030464474 | 2.3701312266366963 |*
*
*
*
Etoposide [μM]
Paclitaxel [μM]
The estimation of cell death was conducted using ApoTox-Glo™ Triplex Assay (Promega) and the extent of dead cells was quantified based on the activity of extracellular dead-cell protease , which converts a kit substrate to fluorescent product. Fluorescence of dead cells was normalized to the fluorescence of viable cells in the culture and is assumed as 1 for untreated control cells.
I-CBP110 (10 μM) was added to cell for 72 h prior to their treatment with anticancer drugs.
Bars in figures represent mean ± SEM. Statistical analysis was conducted using Student t-test and significant changes are marked with * when P < 0.05

## Slide 2
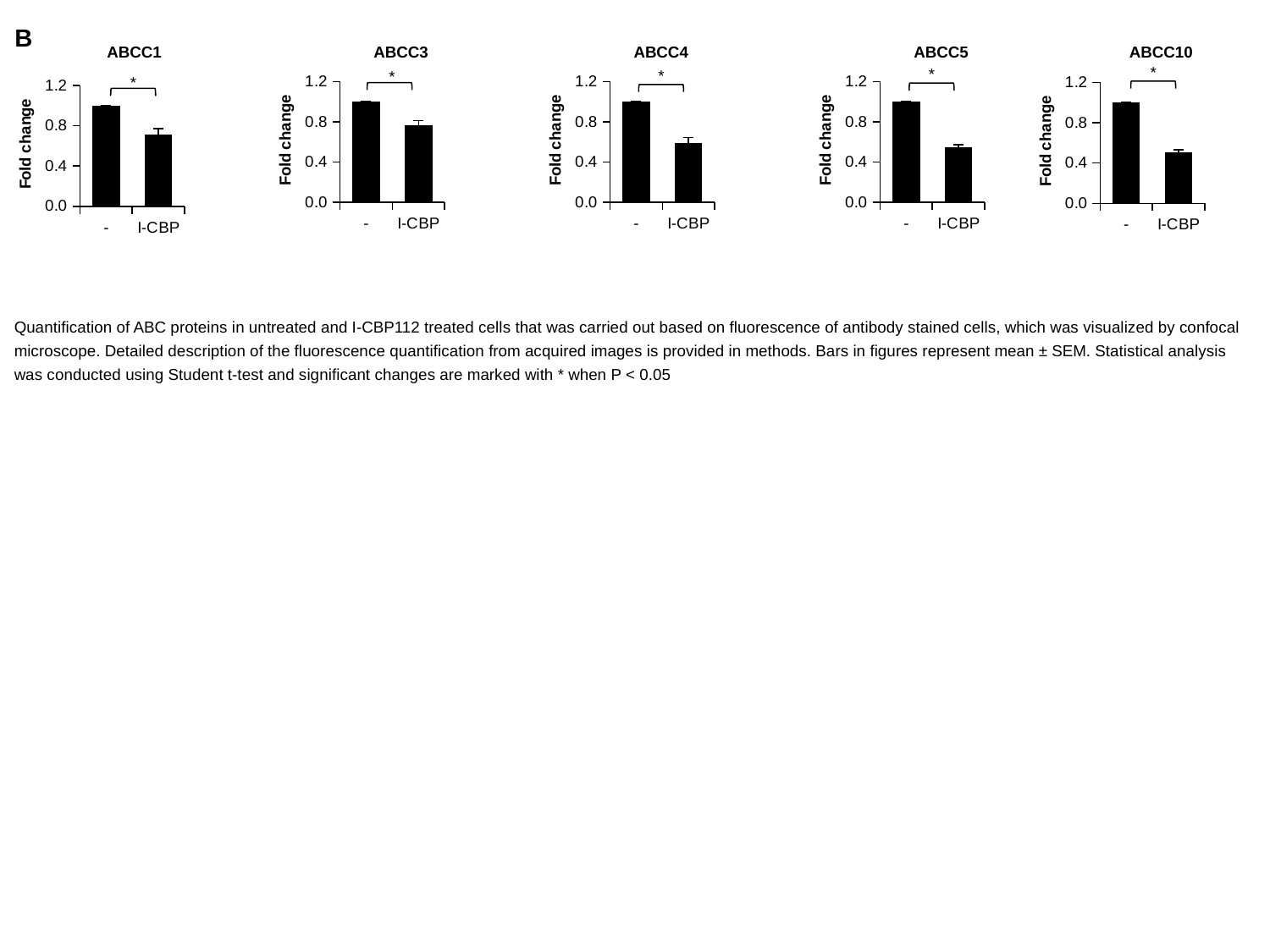

B
ABCC1
ABCC3
ABCC4
### Chart
| Category | |
|---|---|
| - | 1.0 |
| I-CBP | 0.5896882832727464 |*
ABCC5
ABCC10
### Chart
| Category | |
|---|---|
| - | 1.0 |
| I-CBP | 0.7696867004075633 |
### Chart
| Category | |
|---|---|
| - | 1.0 |
| I-CBP | 0.5497560637101355 |
### Chart
| Category | |
|---|---|
| - | 1.0 |
| I-CBP | 0.5088013260282728 |
### Chart
| Category | |
|---|---|
| - | 1.0 |
| I-CBP | 0.7141627708600136 |*
*
*
*
Quantification of ABC proteins in untreated and I-CBP112 treated cells that was carried out based on fluorescence of antibody stained cells, which was visualized by confocal microscope. Detailed description of the fluorescence quantification from acquired images is provided in methods. Bars in figures represent mean ± SEM. Statistical analysis was conducted using Student t-test and significant changes are marked with * when P < 0.05

## Slide 3
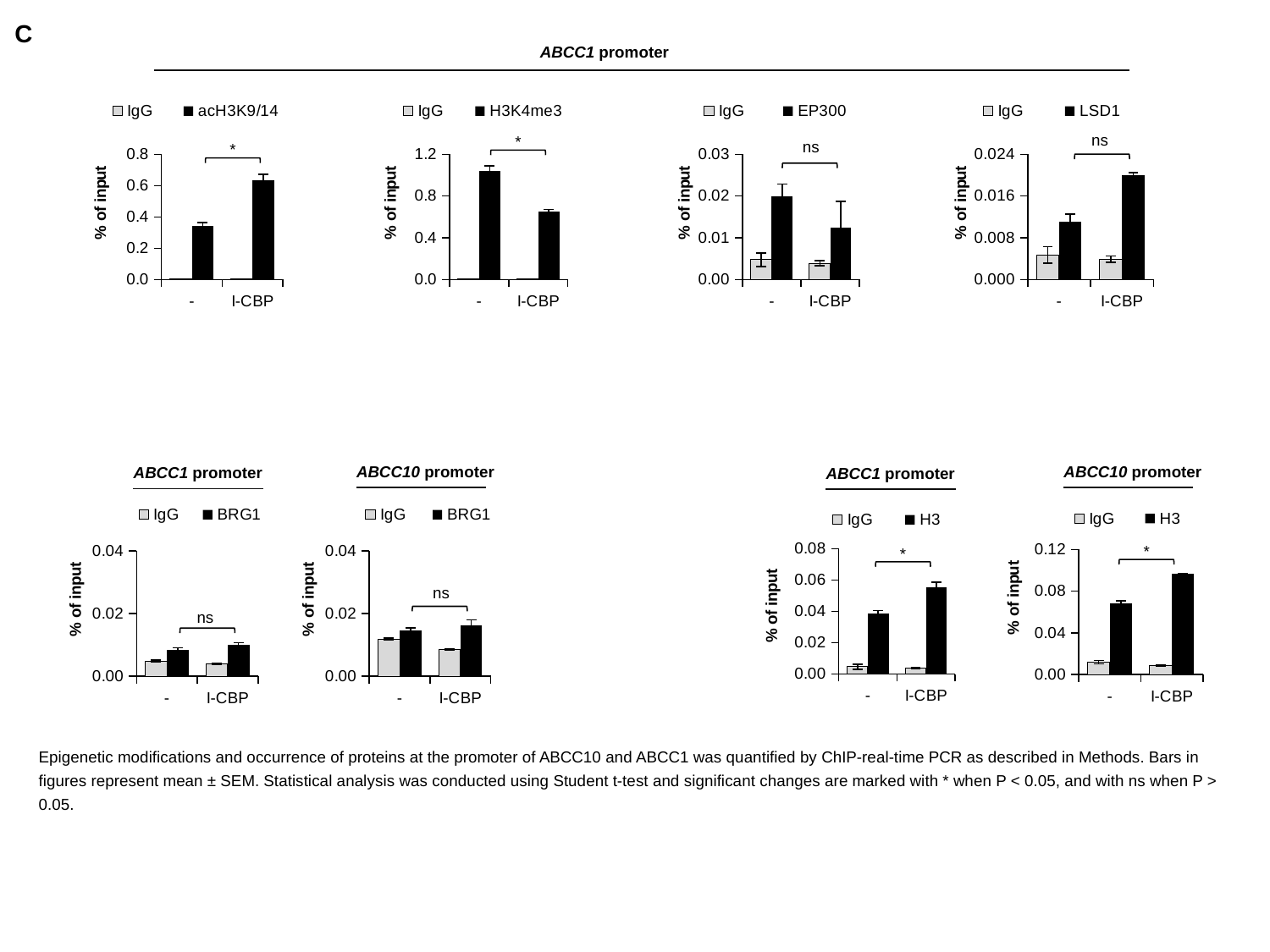

C
ABCC1 promoter
### Chart
| Category | | |
|---|---|---|
| - | 0.004730149368130682 | 0.3402929299860333 |
| I-CBP | 0.003911023519072115 | 0.6331648178633752 |
### Chart
| Category | | |
|---|---|---|
| - | 0.004730149368130682 | 1.0370716662491675 |
| I-CBP | 0.003911023519072115 | 0.6531153566469291 |
### Chart
| Category | | |
|---|---|---|
| - | 0.004730149368130682 | 0.01996621284827648 |
| I-CBP | 0.003911023519072115 | 0.01235046364135042 |
### Chart
| Category | | |
|---|---|---|
| - | 0.004730149368130682 | 0.011134712583964994 |
| I-CBP | 0.003911023519072115 | 0.02002528075149073 |ns
*
ns
*
ABCC10 promoter
ABCC1 promoter
### Chart
| Category | | |
|---|---|---|
| - | 0.004730149368130682 | 0.00830383989217579 |
| I-CBP | 0.003911023519072115 | 0.009947185566671127 |
### Chart
| Category | | |
|---|---|---|
| - | 0.0118193443953483 | 0.014639889593260385 |
| I-CBP | 0.008571881593582863 | 0.016306429310361456 |ns
ns
ABCC10 promoter
### Chart
| Category | | |
|---|---|---|
| - | 0.0118193443953483 | 0.0686057263448014 |
| I-CBP | 0.008571881593582863 | 0.09682530914006712 |ABCC1 promoter
### Chart
| Category | | |
|---|---|---|
| - | 0.004730149368130682 | 0.038757545503040766 |
| I-CBP | 0.003911023519072115 | 0.05561776780846753 |
*
*
Epigenetic modifications and occurrence of proteins at the promoter of ABCC10 and ABCC1 was quantified by ChIP-real-time PCR as described in Methods. Bars in figures represent mean ± SEM. Statistical analysis was conducted using Student t-test and significant changes are marked with * when P < 0.05, and with ns when P > 0.05.
