# Supplementary figures and images for: CBP/p300 Bromodomain Inhibitor–I–CBP112 Declines Transcription of the Key ABC Transporters and Sensitizes Cancer Cells to Chemotherapy Drugs
Source: Cancers (Basel). 2021 Sep 14;13(18):4614. doi: 10.3390/cancers13184614 (PMC8467251; doi:10.3390/cancers13184614)

## Slide 1
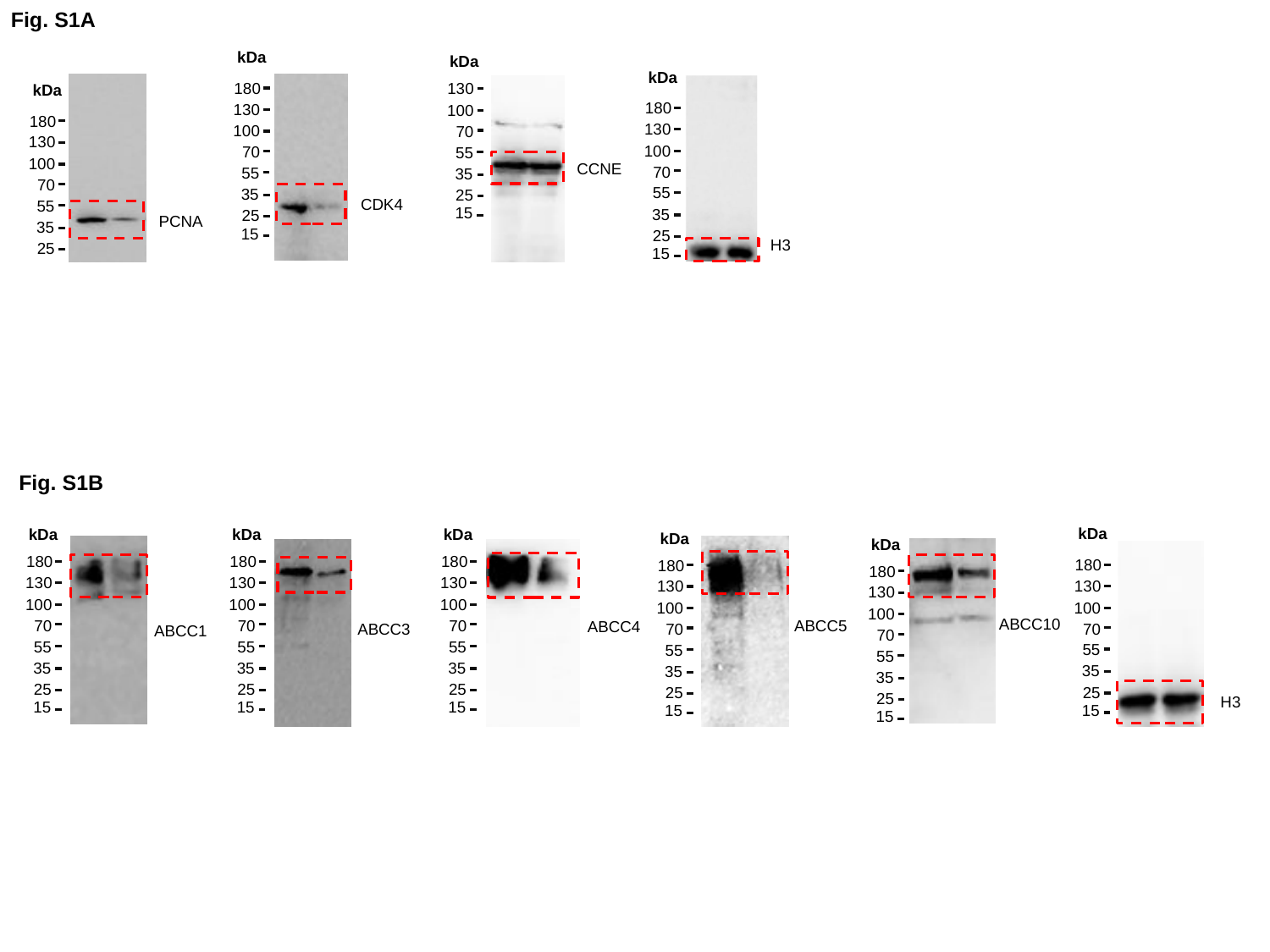

Fig. S1A
kDa
180
130
100
70
55
35
25
15
kDa
130
100
70
55
35
25
15
kDa
180
130
100
70
55
35
25
15
kDa
180
130
100
70
55
35
25
CDK4
H3
CCNE
PCNA
Fig. S1B
kDa
180
130
100
70
55
35
25
15
kDa
180
130
100
70
55
35
25
15
kDa
180
130
100
70
55
35
25
15
kDa
180
130
100
70
55
35
25
15
kDa
180
130
100
70
55
35
25
15
kDa
180
130
100
70
55
35
25
15
ABCC1
ABCC5
ABCC10
ABCC3
ABCC4
H3

Supplement: Supplementary file 1 [file cancers-13-04614-s001.zip › Figure S1.pptx]
